# Redefining shared symbolic networks during the Gravettian in Western Europe: New data from the rock art findings in Aitzbitarte caves (Northern Spain)

**DOI:** 10.1371/journal.pone.0240481

**Published:** 2020-10-28

**Authors:** Diego Garate, Olivia Rivero, Joseba Rios-Garaizar, Martín Arriolabengoa, Iñaki Intxaurbe, Sergio Salazar

**Affiliations:** 1 Instituto Internacional de Investigaciones Prehistóricas de Cantabria (IIIPC, Gobierno de Cantabria, Universidad de Cantabria, Santander), Edificio Interfacultativo, Avda. Los Castros s/n, Santander, Spain; 2 Departamento de Prehistoria, Historia Antigua y Arqueología, Universidad de Salamanca, Cervantes s/n, Salamanca, Spain; 3 Centro Nacional de Investigación Sobre la Evolución Humana (CENIEH), Paseo Sierra de Atapuerca s/n, Burgos, Spain; 4 Departamento de Geología, Euskal Herriko Unibertsitatea/Universidad del País Vasco, Barrio Sarriena s/n, Leioa, Spain; Max Planck Institute for the Science of Human History, GERMANY

## Abstract

The renewal of the archaeological record, mainly through the discovery of unpublished sites, provides information that sometimes qualifies or even reformulates previous approaches. One of the latter cases is represented by the three new decorated caves found in 2015 in Aitzbitarte Hill. Their exhaustive study shows the presence of engraved animals, mainly bison, with formal characteristics unknown so far in the Palaeolithic art of the northern Iberian Peninsula. However, parallels are located in caves in southern France such as Gargas, Cussac, Roucadour or Cosquer. All of them share very specific graphic conventions that correspond to human occupations assigned basically to the Gravettian cultural complex. The new discovery implies the need to reformulate the iconographic exchange networks currently accepted, as well as their correspondence with other elements of the material culture at the same sites. Thus, we have carried out a multiproxy approach based in statistical analysis. The updated data reveals a greater complexity in artistic expression during the Gravettian that had not been considered so far, and also challenges the traditional isolation that had been granted to Cantabrian symbolic expressions during pre-Magdalenian times.

## Introduction

**Fig 1:**
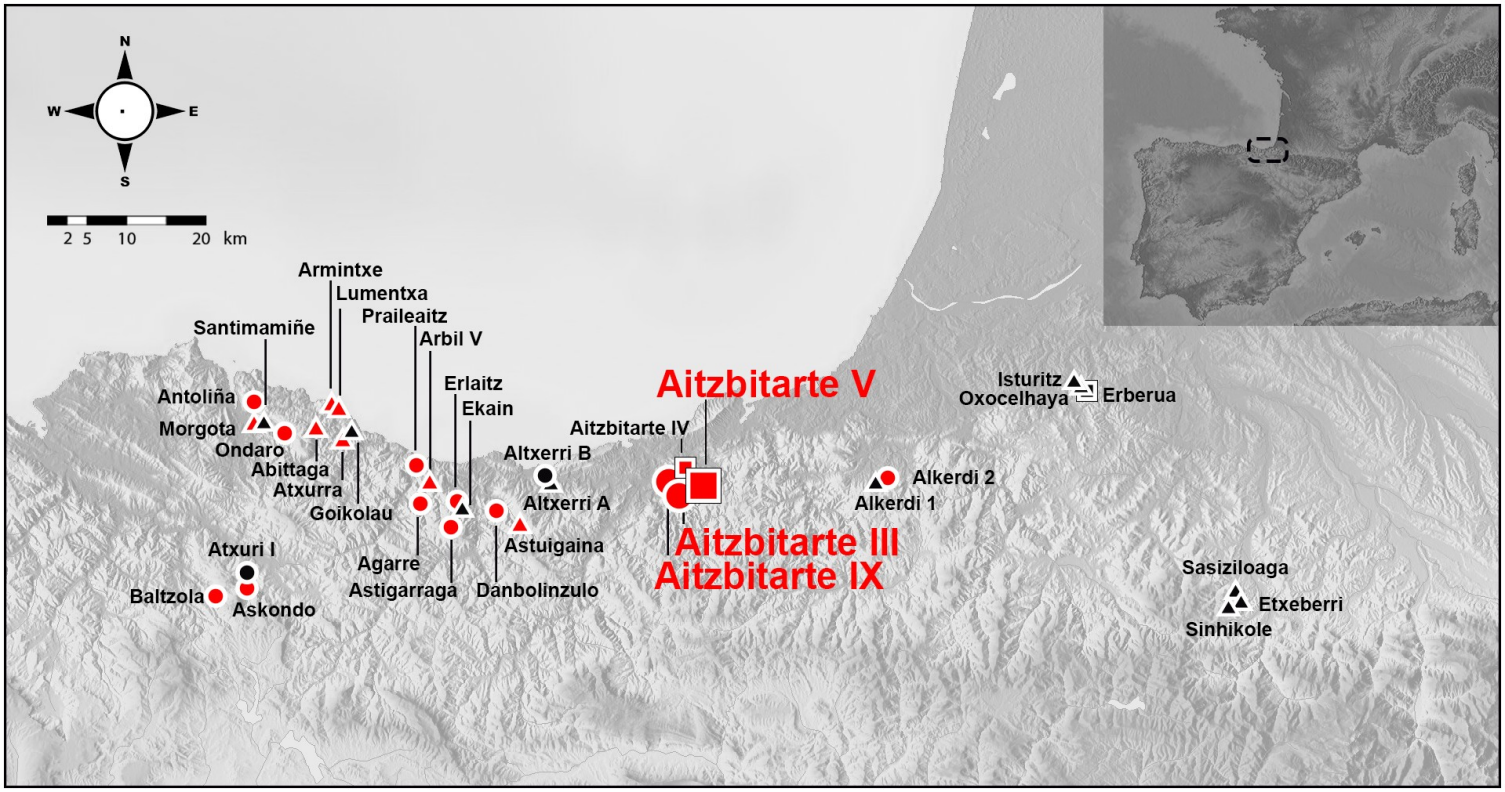
Decorated caves in Eastern Cantabrian Region (from Nervión to Bidasoa) and the Western Pyrenees (from Bidasoa to the Gaves de l’Adour) (base map: https://maps-for-free.com): Black discovered during the 20th century; red found in the 21st century; circle for pre-Magdalenian ensembles; triangle for Magdalenian ones; square for complex ensembles (pre-Magdalenian and Magdalenian).

In recent years, research on Palaeolithic parietal art in the north of the Iberian Peninsula has experienced a remarkable renewal in activity, especially in the Eastern Cantabrian Region (the area covering from the Nervión river to the Bidasoa). In this area, the density of decorated caves used to be significantly lower than in surrounding regions (the rest of the Cantabrian Region, the Northern Pyrenees and Périgord) despite its geostrategic position in the centre of those regions and in the contact between the Iberian Peninsula and the rest of Europe. As a consequence of this new research, the number of known parietal ensembles has increased threefold, from about ten to over thirty. Albeit the new sites with larger assemblages, such as Atxurra, Aitzbitarte IV or Armintxe (Fig 1), are dated to the Magdalenian, a considerable proportion of the newly discovered rock art sites (n = 14) can be attributed to periods previous to the Magdalenian -absent so far in the area-, therefore filling not only a geographical gap but also a chronological one [1].

In this regard, the discoveries made in Aitzbitarte Hill in 2015 are of special interest. In three caves, Aitzbitarte III, V and IX, fine engravings of animal figures correspond to an artistic style that was unknown in the northern Iberian Peninsula. In contrast, the closest parallels of these figures are found in caves in south-west France that were decorated during the Gravettian cultural complex, such as Gargas, Cussac and Cosquer [2]. That is, as we will see, all these sites are sharing the same graphic conventions especially developed during the Gravettian and, possibly, extending over time. The new findings that we present below, as well as the revision of the whole of the archaeological record, allow us to resize or even redefine, the area of influence of the conventions shared by the decorated caves of the Gravettian period.

The Gravettian is a pan-European cultural complex, but with an unequal distribution across Europe [3]. Also, the Gravettian covers a large time period of *ca*. 10.000 years (*ca*. 34,000 to *ca*. 24,000 cal BP) [4–6]. As a result, the Gravettian cultural complex displays a great chrono-spatial variability, which has promoted the definition of facies, each with its own characteristics [7]. In western Europe, the Gravettian has been divided into an initial Gravettian (Fontrobertian or Bayacian), a middle Gravettian (Noaillian or Rayssian) and the late Gravettian or proto-Magdalenian. Some scholars now question the unity of the Gravettian and propose the existence of different cultural complexes, with their particular traits and evolution [5]. For example, it has been hypothesised that the Noaillian originated from the Late Aurignacian and persisted over a long period in the Franco-Cantabrian area [8]. In any case, there is a generalization and uniformity in the symbolic realm. For example, mortuary practices have been identified with an unprecedented record in terms of the number of individuals and associated artefacts [9]. The decorated Gravettian ensembles, particularly in the field of cave art, constitute a quite well-known and well-dated group, since at least fifteen sites have either direct dates (for drawings or associated archaeological remains) or decorated objects in dated stratigraphy [10]. Stencilled hands in parietal art and the famous ‘Venuses’ in the portable modality are the best-known points of reference of Gravettian art, but not the only ones [11]. In south-west France, the very specific graphic production is characterised by the absence of perspective in animal representations, which are mostly engraved and also follow very particular graphic conventions [12], as will be explained below. Their definition has been supported by new discoveries of parietal art in Cussac [13] and Cosquer [14], and of portable art at Isturitz [15]. Some researchers have noted the similarities between those ensembles and such sites as Gargas, Roucadour and Pech Merle, particularly in characteristic motifs (female figures and handprints) and some specific conventions (mammoth tusks, quadruped limbs in frontal view, etc.) [16, 17].

In the present paper, the study of the new decorated caves of Aitzbitarte together with the reappraisal of other Gravettian parietal and/or portable ensembles, has enabled us to discuss about the iconographic exchange networks and of the connections between the populations in the different regions of western Europe during the Gravettian. Our main objective has been to integrate the new findings into a thematic and formal study, based on objective and quantifiable criteria, with the aim of specifying and establishing the distribution areas of the animal conventions characteristic during the period and region referred to.

Some specialists call these exchange networks ‘symbolic territories’: identification or identities shared by human groups, not necessarily demarcated within visible material borders but following some common cultural norms [18, 19]; where the graphic analogies can be distinguished at three geographic levels: local, regional and supra-regional [20, 21]. In our case, we consider that this term -symbolic territories- could conceal greater complexity in the case of hunter-gatherer societies. The complexity of territorial analyses applied to ethnographical hunter-gatherer societies has been outlined and described by several authors [22]. It cannot be limited to formal analogies between territories, especially if it is left out data from the archaeological record, as; economic resource procurement, the seasonality of the sites or the paleo-diet [23, 24]. We therefore prefer to speak about networks and areas of influence, rather than frontiers and territories, which are concepts more closely related to sedentary societies.

## Materials and methods

### Aitzbitarte caves

**Fig 2:**
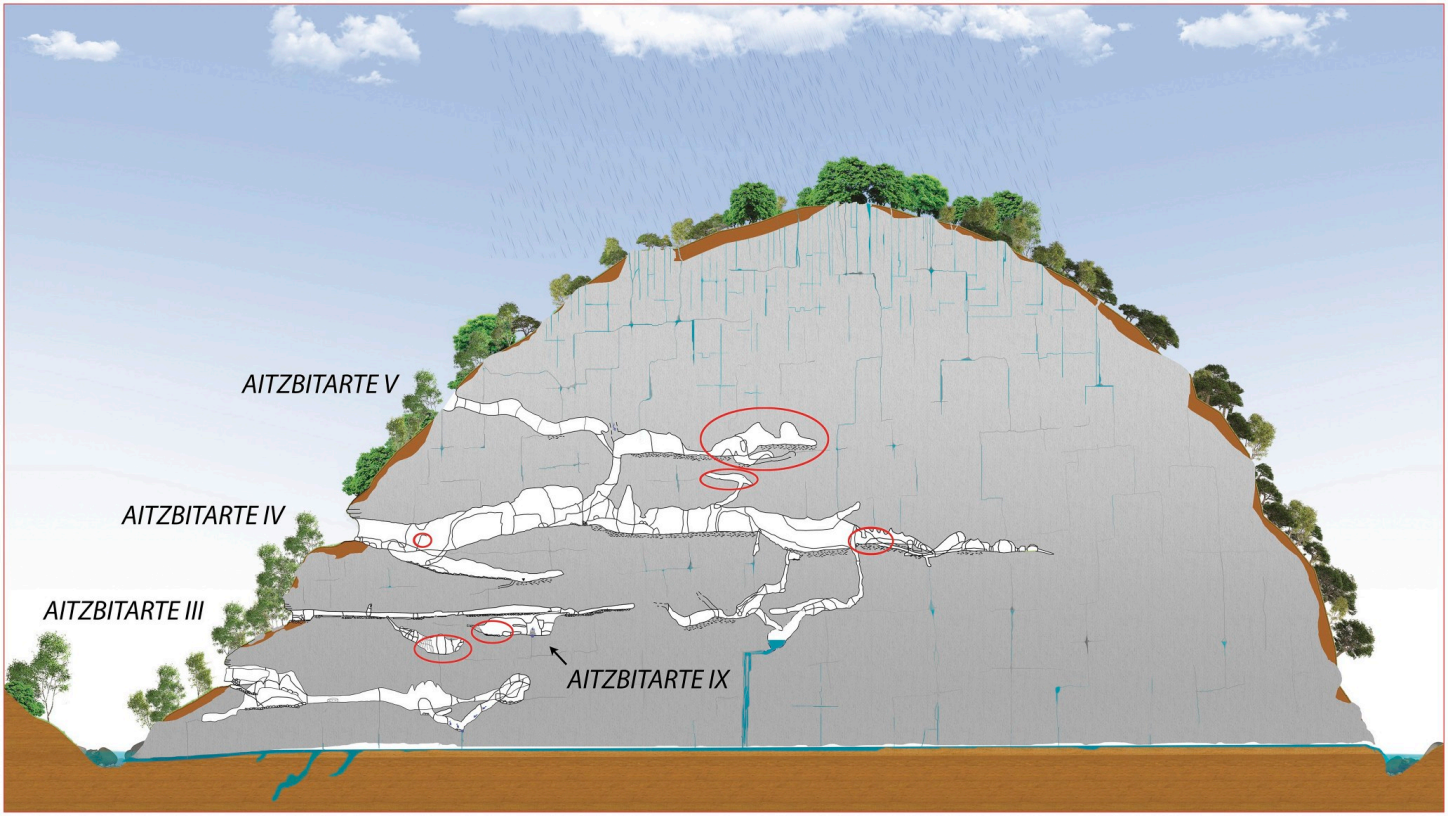
Cross-section though Aitzbitarte hill showing the main speleological phenomena. The decorated sectors are indicated with a red circle (Felix Ugarte Elkartea).

The Aitzbitarte hill is located in the easternmost part of the Cantabrian Region, in a tributary valley of the Urumea basin close the foothills of the Pyrenees and the border between Spain and France, that is, between the Iberian Peninsula and the rest European continent [ETRS89 UTM zone 30N x: 589639 y: 4790595 z: 275]. This limestone hill has been subjected to powerful karst processes that have created numerous caves and shafts, and some of them preserve archaeological records from the Upper Pleistocene to the Holocene [25]. The entrances of the caves with archaeological deposits are located on the western side of the hill (Fig 2), about 40 m above the current stream that flows below them. Those karst features have formed in the Upper Albian bioclastic calcarenites, where the fall in phreatic level and variation of sedimentation in the caves have allowed the formation of horizontal passages and cave systems on different levels.

First archaeological explorations in Aitzbitarte hill were made at the end of XIX century, but the first scientific excavations were made long after (1960 to 1964) in Aitzbitarte IV by J.M. Barandiarán [26]. In the 1960’s a small archaeological deposit in Aitzbitarte V was discovered in the entrance of the cave [27]. Further on, between 1985 and 2002, J. Altuna excavated Aitzbitarte III [28, 29]. The first evidence of Palaeolithic parietal art in these caves, a series of shapeless remains of red pigment, perhaps with a red zoomorphic, was found in Aitzbitarte IV in 2012 by D. Garate and J. Rios-Garaizar [30].

In Aitzbitarte Cave III there are two excavations areas, one close to the entrance and the other one situated a further inside the cave. The sequence in the entrance revealed a succession of Middle Paleolithic, Late Aurignacian, Early Gravettian (Noaillian), Late Gravettian (also Noaillian), and a mix of Solutrean and Magdalenian [31]. The sequence in the interior shows a succession of Middle Gravettian (Noaillian), Late Gravettian (also Noaillian), and possible Badagoulian/Early Magdalenian [32]. According to new dates, the Gravettian at Aitzbitarte III started very early (*ca*. 36,000 cal BP) [6], and probably lasted until *ca*. 22,000 cal BP. This Gravettian characterized by lithic assemblages dominated by blade products and Noailles burins; typical bone tools as the Isturitz type points [33, 34]. Interestingly, no portable art has yet been identified in the Gravettian units, contrasting with the Noaillian from Isturitz [15]. The faunal assemblages dominated in the entrance sector by bovids (*Bos/Bison*), red deer and chamois, and by *Bos/Bison* in the interior [35, 36]. In these assemblages, the presence of consumed birds, fish and molluscs has also been attested [28, 29]. In Aitzbitarte V only few, probably postpaleolithic, artefacts were recovered in a small survey at the entrance, and up to now no excavation has been conducted in Aitzbitarte IX (undoubtedly because the original entrance is completely collapsed during its first 15 meters).

### Archaeological survey and rock art recording

**Fig 3:**
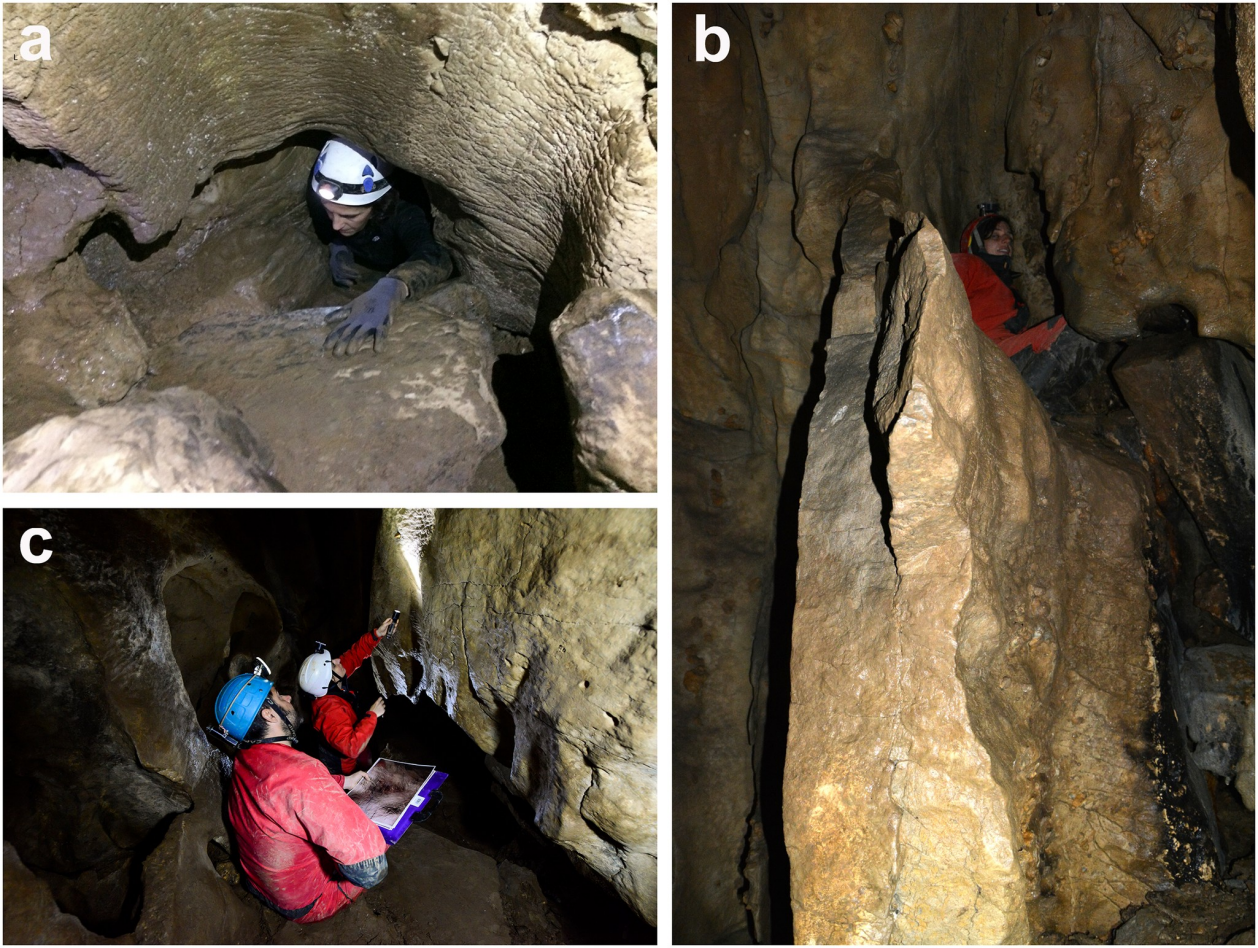
Working conditions in the decorated passage in Aitzbitarte Cave III: a) Access to the sink-hole leading to the passage; b) descent to the decorated area; c) archaeological recording process of the engraved walls (S. Laburu).

In September 2015 figures of bison engraved in a Palaeolithic style were discovered in Aitzbitarte V by a team lead by D. Garate. One week later, two spelunker collaborators, J. Busselo and G. Studer, found more representations in Caves III and IX [2]. The three caves were exhaustively explored and documented in 2016 under the authorization and funding of the local administration (Diputación Foral de Gipuzkoa), in charge of this archaeological heritage. The narrowness of the cave passages, the poor state of conservation of the walls, and the type of engravings difficult the analysis and demanded the implementation of different technological solutions to overcome the difficulties (Fig 3). Given that reflectance transformation imaging or laser scanning techniques were not applicable in rock art recording, due to the narrowness of the spaces and the difficulties to access some sectors, graphic documentation was based on the application of three-dimensional restitution techniques (close-range photogrammetry) combined with systems for the digital treatment of images and graphic restitution [37–40]. Specifically, the near object photogrammetry has been carried out using grazing lighting, which facilitates the identification of the engravings on the obtained textures as well as orthorectified images of the panels. This methodology is not without difficulties since the artificial lighting used (flash), necessary for a correct visibility of the engravings, makes it difficult to create photogrammetric models. Despite this, the results obtained are optimal for the documentation of the engraving, as well as for the restitution of the supports.

### Data analysis

In order to perform formal analysis, a series of attributes-values has been established, defining the formal characteristics of the figurative motifs. These attributes refer to certain conventions in the representation of limbs and horns as explained below.

Factorial Correspondence Analysis (FCA) [41] has been chosen for this study as it is particularly useful for the analysis of the scores. This method of analysis has many advantages; the objects that look alike are located near to each other, while those that differ are separated. By virtue of the principle of duality between objects and properties, the two series of points are correlated and may be placed in the same graph. Objects and properties that are frequently associated are close together and two properties are close if they appear often associated with a single individual [42].

In this case, the analysis has been carried out using a Burt Table: a symmetrical matrix whose diagonal contains the total number of frequencies of all the analysed categories while the spaces divided by this diagonal contain the total number of associations crossed between each of the categories of the different variables [43]. The analysis was carried out in this way based solely on the property values, without taking the objects into account. FCA has been used in various archaeological domains and has proved to be particularly useful in the case of artistic items, when the objective was to correlate formal features with the human groups who produced them in order to study their differences, degree of independence and relationships between them [42, 44–49].

This method was complemented with Ascending Hierarchical Clustering (AHC) that allows progressive grouping of elements in classes based on a measure of ‘affinity’ or proximity. The result is a treelike hierarchical classification or *dendrogram*. Finally, the correlation between the criteria was analysed with the Z-test [50]. Only probabilities higher than 95% were retained as significant in the discussion.

## Results and discussion

Between 2016 and 2018 we have carried out field research in the Aizbitarte caves under the authorization and funding of the *Diputación Foral de Gipuzkoa* (Spain), in order to document the rock-art evidences.

### Aitzbitarte III

**Fig 4:**
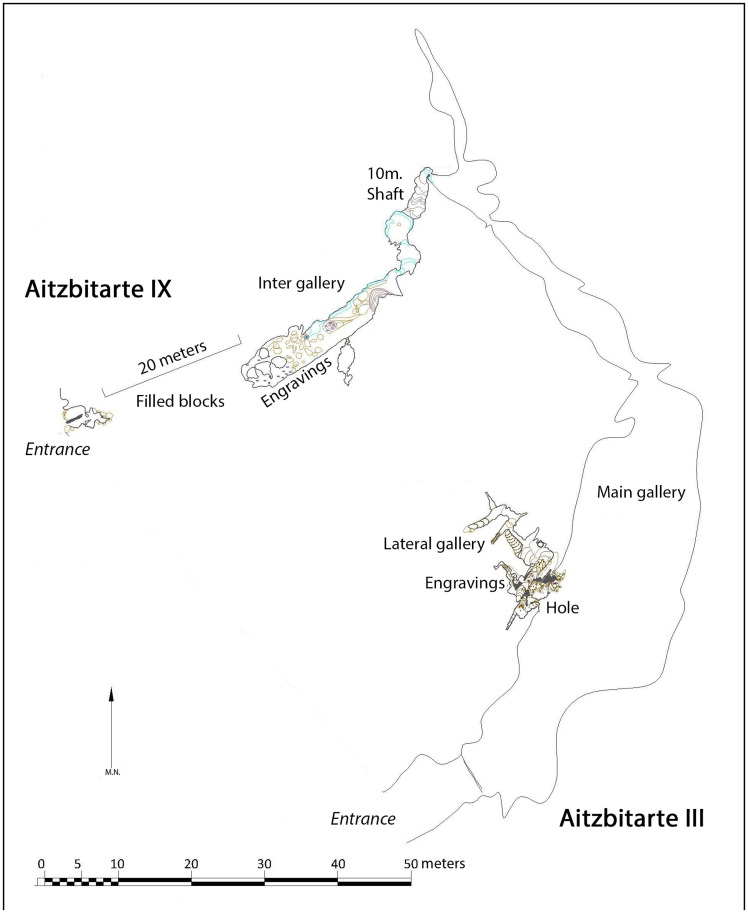
Plan of Aitzbitarte Caves III and IX. The decorated areas are indicated (after a survey by the Felix Ugarte Elkartea group).

The entrance of Aitzbitarte Cave III is 7 x 2 m in size, from where the cave continues along a low horizontal passage that forms one of the levels in the system. This passage is relatively wide (but with low-ceilings) for about 100m, and then becomes gradually narrower until it ends in a 10m-shaft to Aitzbitarte Cave IX (Fig 4) which belongs to the lower level of the system. The floor of the main passage is formed by fine sediments and is more or less smooth and flat along its length, with no significant speleothems or side-passages. On one side of the passage, the fluvio-karst sediments practically reach the roof, and these represent the fluvial and paragenetic phase of the passage.

On the left-hand wall, between the two excavation areas with Gravettian layers, a small hole at floor level leads to a vertical 1 x 1 m tube connecting with a very narrow maze of passages. The access is blocked by *Buntsandstein* sandstone boulders cemented by a thin layer of calcite. This is quite porous and contains fine detritus, and diagenetic crystal fabrics. Because of this, the samples taken for U/Th dating in the CENIEH Uranium Series Laboratory (Spain) did not display the necessary conditions to function as a closed system and the results of the analysis have not been conclusive (S1 File). This passage has acted as a sink-hole where infiltration water accumulated, and this created an extremely damp and muddy environment. It was originally a descending passage that connected the different levels in the cave system. However, because of the dating issues it has not been possible to determine exactly when the passage became partially blocked by the boulders.

**Fig 5:**
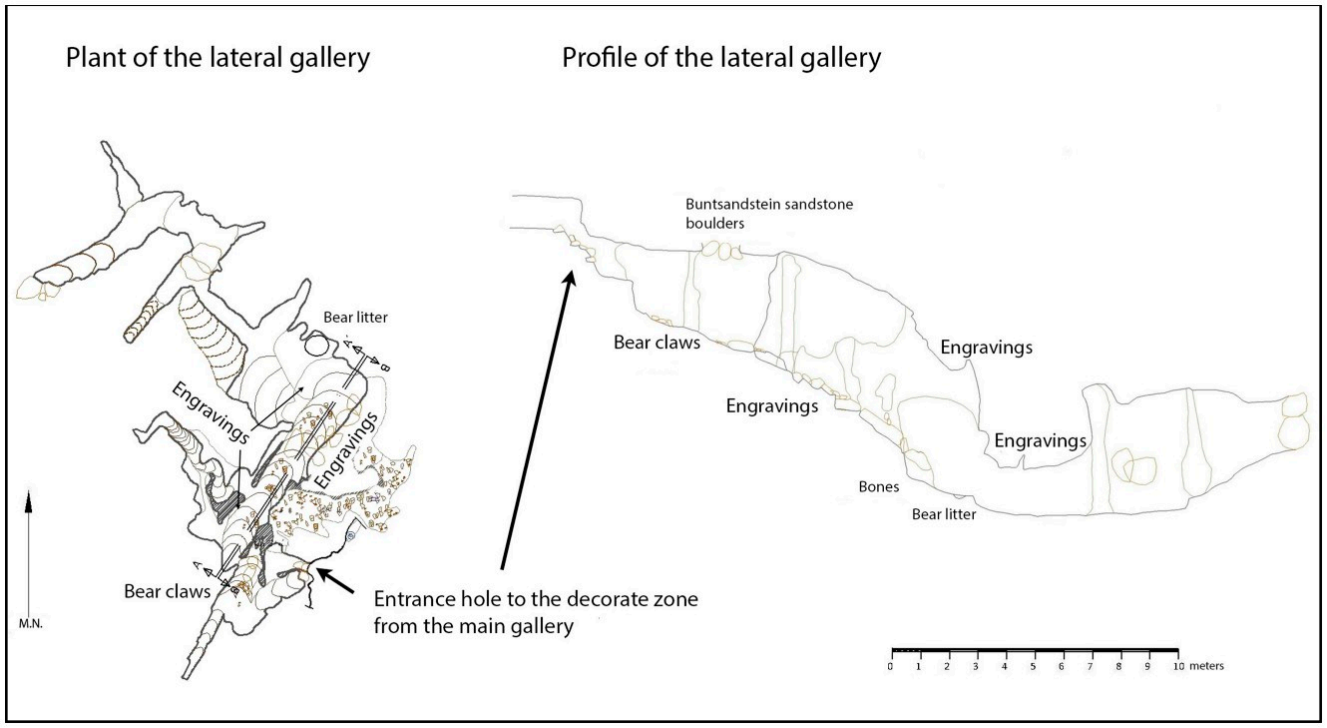
Plan of the decorated side-passage in Aitzbitarte Cave III. The decorated areas are indicated (J. Busselo).

The first part of the lower level of passages consists of a very narrow and steeply-sloping passage (Fig 5) with small alcoves on each side. Eleven decorated panels have been identified in these spaces (seven on the right-hand wall and four on the left-hand wall), over a distance of 10 metres. The height above the floor of these panels increases as the passage descends, from about 1.5 m at the start to 3 m in the case of the last representations.

**Fig 6:**
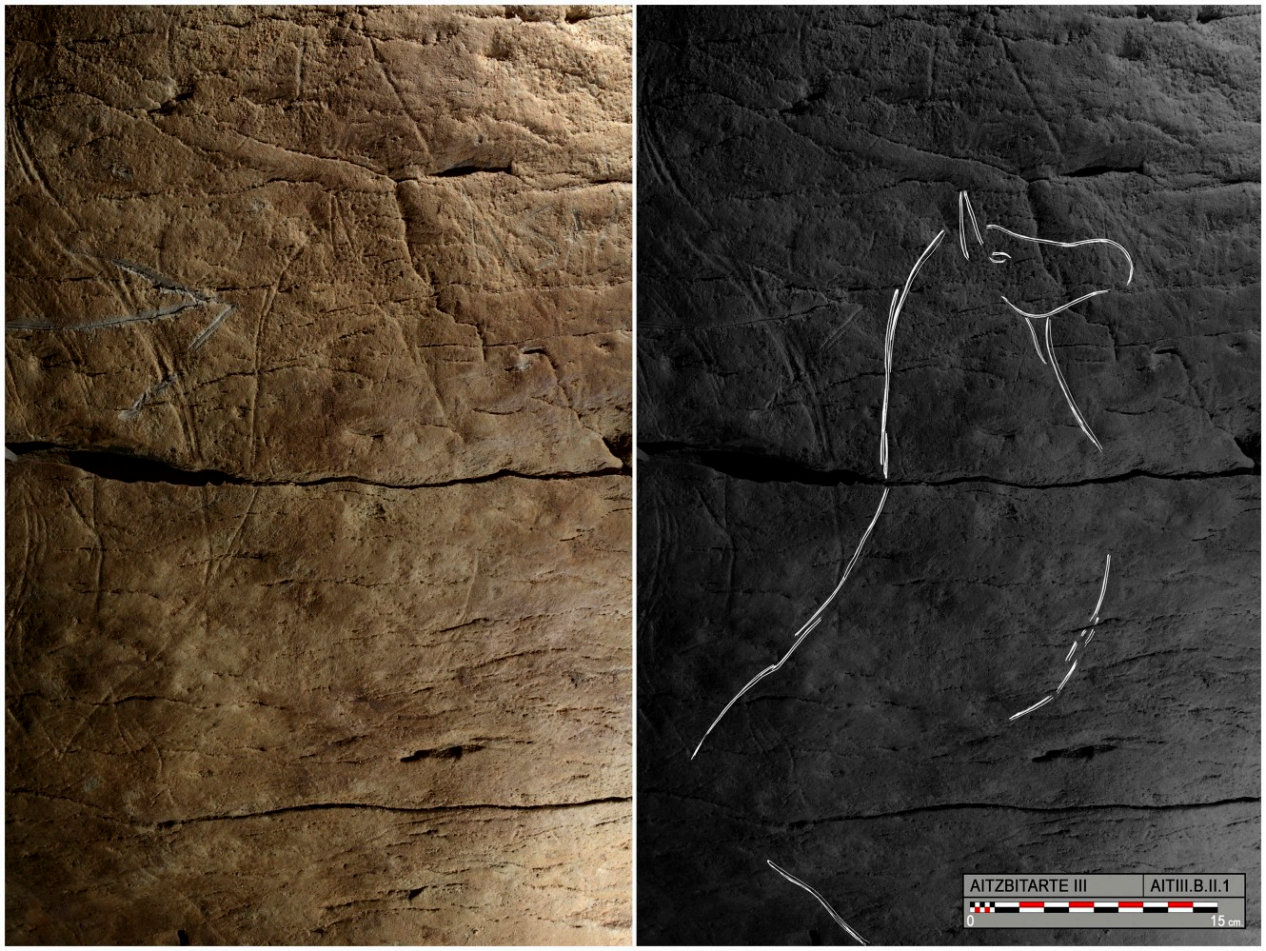
Photograph and tracing of horse B.II.1, engraved on the right-hand wall in Aitzbitarte Cave III (O. Rivero and D. Garate).

At the start of the ramp, on the right-hand wall, a panel (B.II) is badly damaged by fresh clay and modern graffiti. Several representations can be seen under them, especially a complete horse (B.II.1) facing towards the entrance and oriented upwards (Fig 6). It has a stepped mane, an eye, a sinuous jaw and pronounced belly. Opposite it, superimposed, and facing towards the interior, a headless bison (B.II.3) consists of the hind legs, the rump indicating the inguinal, tail and back. Continuing down the ramp, some isolated bison horns are more or less individualised, oriented towards the exterior (B.III.1) and then a more complete depiction of the same animal (B.IV.1) includes the legs in two planes, and the horns in correct perspective.

**Fig 7:**
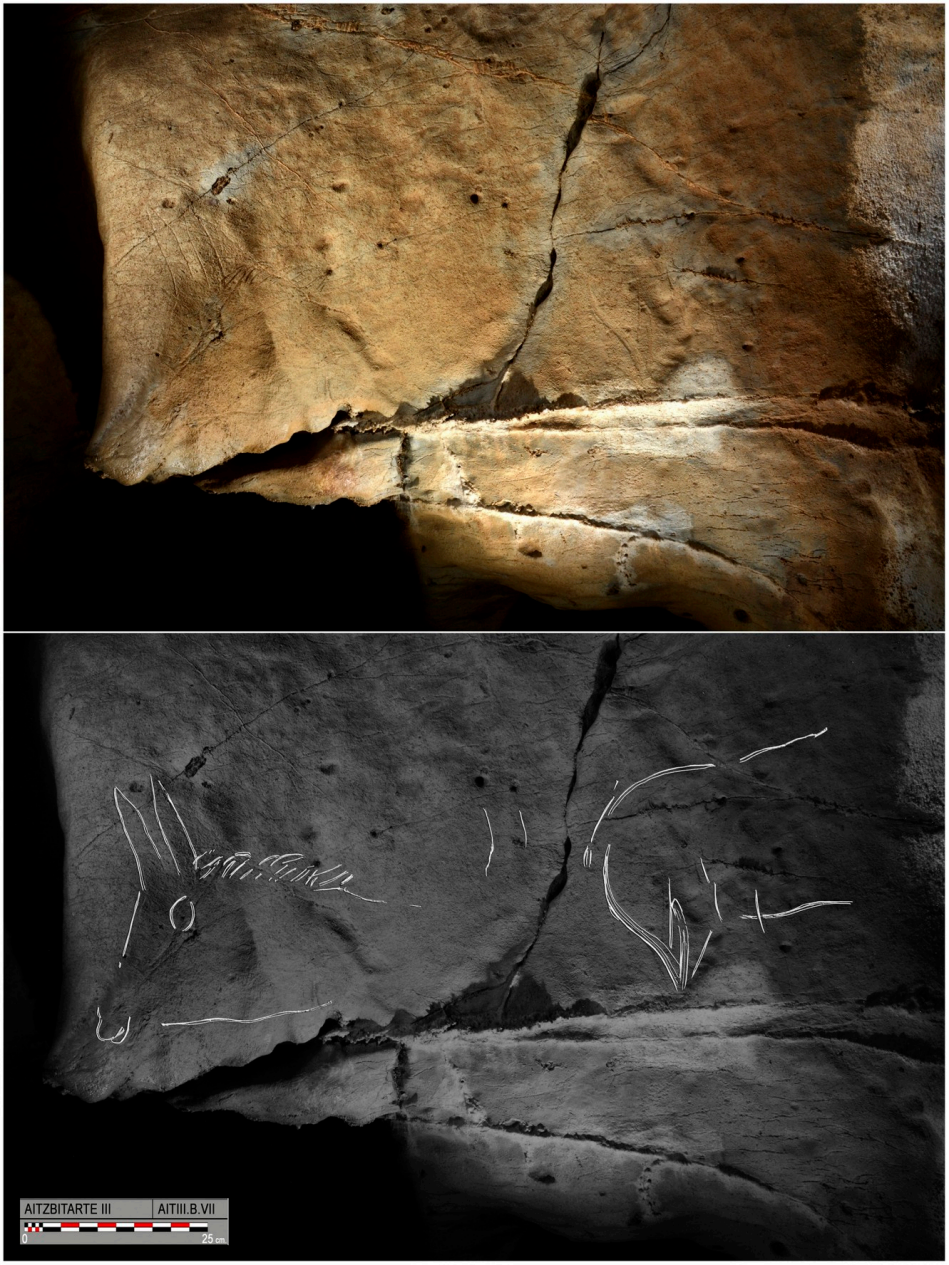
Photograph and tracing on panel B.VII at the bottom of the ramp in Aitzbitarte III: Indeterminate animal B.VII.1; bovid horns B.VII.2; and horse fore-quarters B.VII.3 (O. Rivero and D. Garate).

In an alcove nearly at the end of the ramp, on a flat surface at floor level (B.V), a group of engravings are hard to interpret but include a possible representation of an ibex and some bison horns. The passage then turns to the left, leaving an overhanging section of wall opposite the ramp. Here an animal (B.VII.1), represented with wide engraved lines, consists of a short tail and voluminous hind-quarters but without the fore-quarters. To its left, two horns (B.VII.2) are depicted in perspective and next to it, the fore-quarters of a horse (B.VII.3) is adjusted to a pointed edge of rock which acts as its muzzle. It is formed by the mane represented with disorderly lines, the ears with very long double lines, an eye and sinuous jaw (Fig 7).

**Fig 8:**
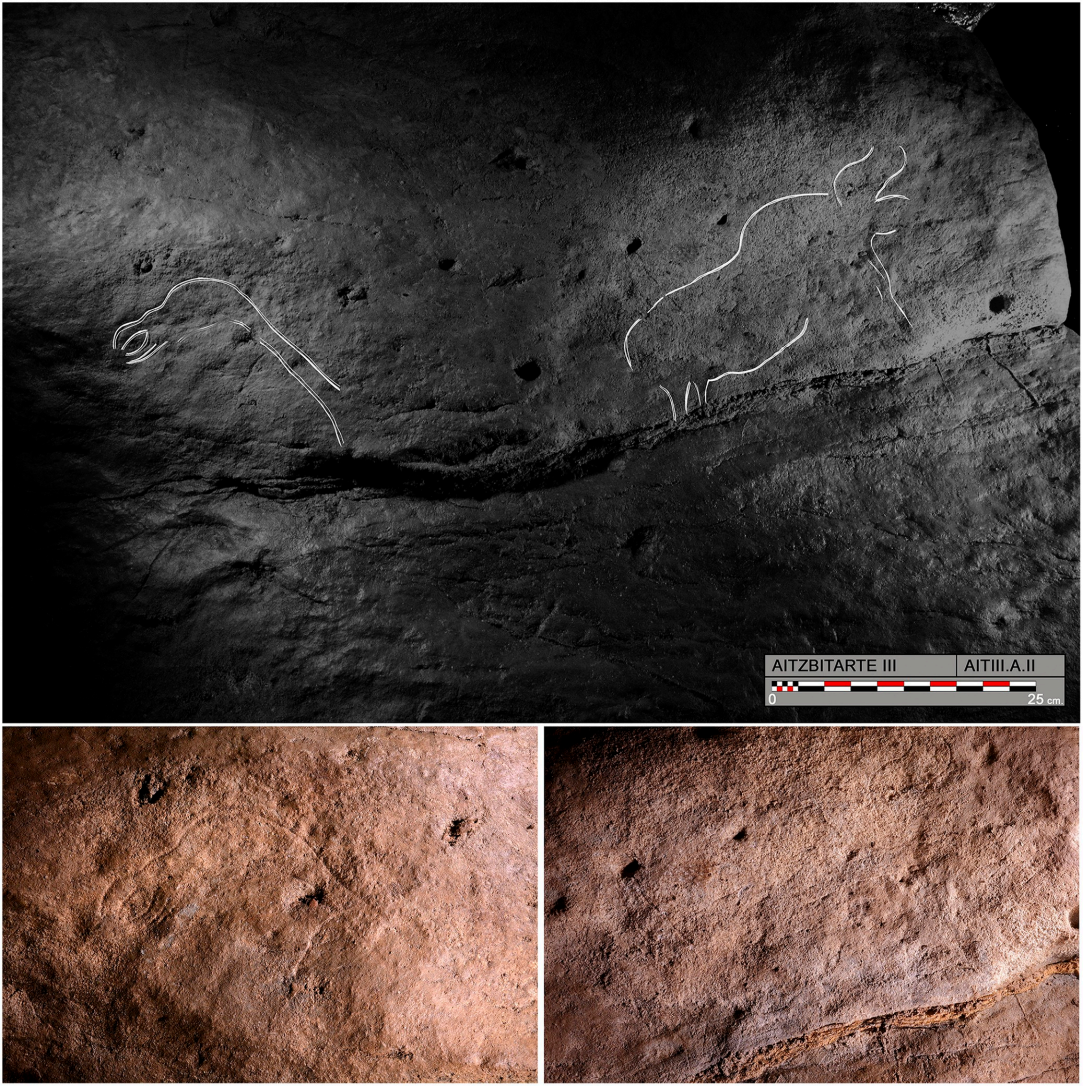
Engravings in the A panel of Aitzbitarte III. Top: Tracing of the panel formed by the bird A.II.2 and the bison A.II.1, engraved on the left wall. Bottom left: Detail of the bird’s head. Bottom right: Detail of the bison (O. Rivero and D. Garate).

On the left-hand wall, a first panel (A.I) displays several cervical-dorsal lines and bison horns that are very poorly preserved owing to visitors rubbing against them. It is followed by a second panel (A.II) at floor level with a covering of calcite on its right (Fig 8, top) which contains a representation of a bird (A.II.2) formed by its head and a long slender neck (Fig 8, bottom left). It is next to a small finely-engraved complete bison (A.II.1) with its horns without perspective and its legs in a single plane (Fig 8, bottom right). These two figures face in opposite directions.

**Fig 9:**
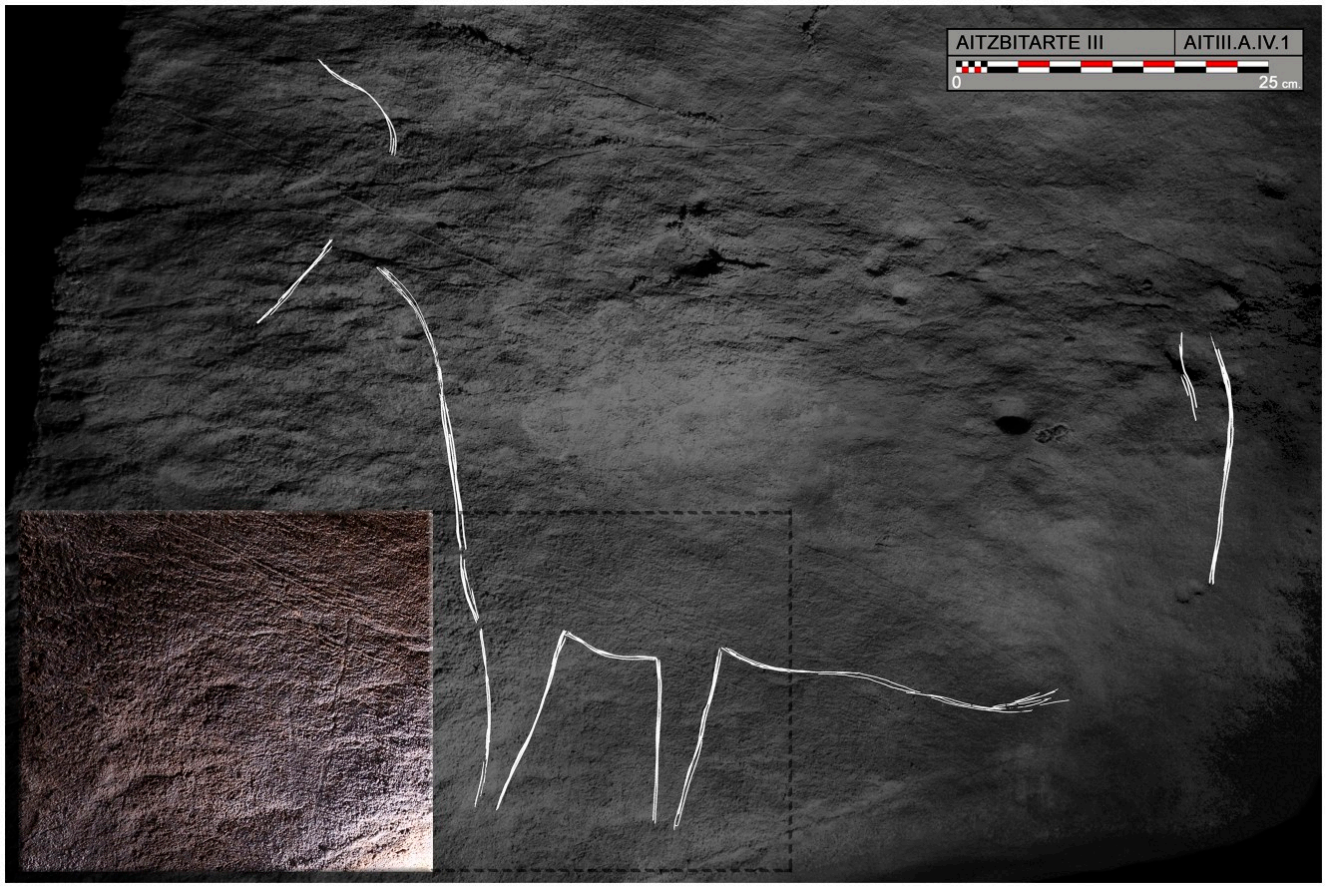
Tracing and detailed photograph of the front legs of the aurochs A.IV.1, engraved at the end of the left-hand wall in Aitzbitarte III (O. Rivero and D. Garate).

A third panel (A.III) is badly damaged, but two horses (A.III.1 and A.IV.3) can be made out, engraved on the rubbed surface. At considerable height because of the decreasing floor height, two aurochs (A.III.4 and A.IV.1) are represented as fore-quarters facing each other. The latter of these displays very detailed hooves whereas the opposite figure’s legs are shown as a frontal view (Fig 9). This figure is at a height of 3m above the ramp, in a place with very difficult access, and it is probably for that reason that the upper part and hind-quarters were not completed.

### Aitzbitarte V

**Fig 10:**
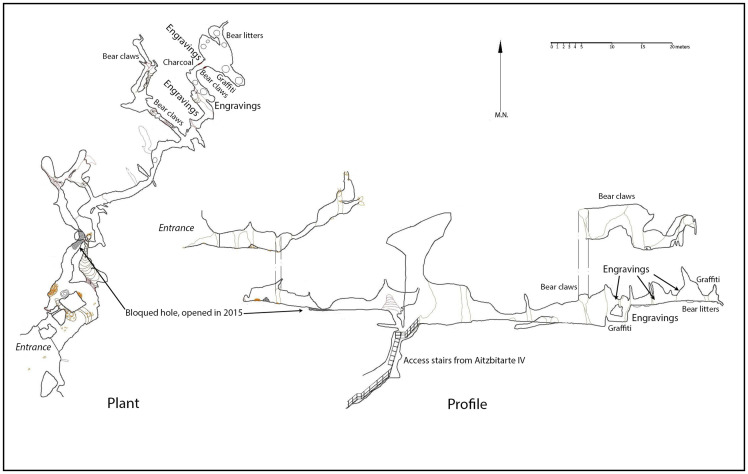
Plan of Aitzbitarte Cave V. The decorated areas are indicated (J. Busselo).

Aitzbitarte Cave V is located about 20m above the other archaeological sites (Caves III, IV and IX) on the same western side of the hill. It is a shorter cave, with a length of 60m from the entrance to the final chamber. The entrance hall, 10 x 3 m in size, is divided into two side spaces by large collapsed boulders and a narrow passage leads into the interior of the cave. This passage was totally blocked by clay sediment until it was dug out by members of Felix Ugarte Elkartea caving group in 2015, when they found lithic artefacts and pottery attributed to recent prehistory. Before then, the cave could only be entered by climbing a 25 m-high chimney from Cave IV. The inner cave is formed by a straight main passage, between 2 and 4 m in height and an average of 2m in width, with two side passages on the left and, after a corner, a final semi-circular chamber on a higher level 3 m above the passage (Fig 10).

Four sectors with Palaeolithic engravings are located in the final part of the cave. Three of these are attributed to the Gravettian and the other to the Magdalenian [2], following formal conventions (absence of perspective and details in the former and greater naturalism and use of perspective in the latter). The walls have been badly damaged, first by the claw marks of bears that hibernated in the cave and secondly with graffiti left by visitors to the cave during the 20th century.

**Fig 11:**
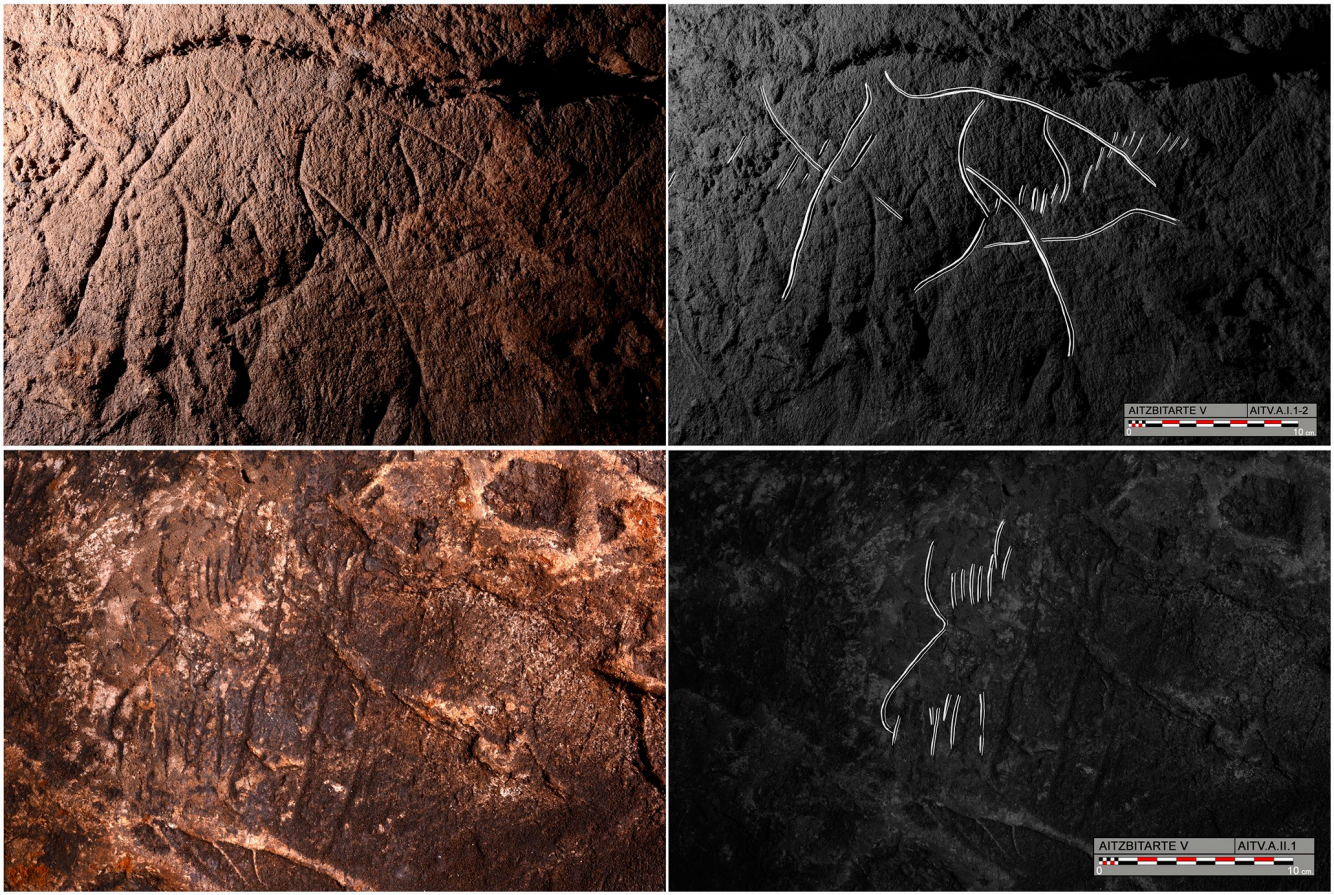
Photograph and tracing of bison A.I.1 and A.II.2, engraved in Sector A in Aitzbitarte Cave V (O. Rivero and D. Garate).

The first decorated sector (A) is located just before the passage turns left and ends in the final chamber. A narrow ledge at a height of 3m acts as a balcony above the contiguous passage. It contains two partial depictions of bovine (probably bison because of the hump and the beard) (A.I.1 and A.II.1), reduced to the head, horns and start of the hump (Fig 11). They both display a horn connected to the forehead and a series of short lines to indicate the hair and hump, which are separated by the other horn. Some other lines next to them do not form figurative depictions. On the other side of the balcony, a narrow passage (B) contains engravings of four bison which will not be considered further here as they have been attributed to the Magdalenian, by means of stylistic features [2]. After, in the left side of a sinuous gallery, there is a small and isolate engraving of a bison which preserves the head, the start of the hump and the two front legs (C.I.1). In the final chamber (D), which is badly deteriorated by graffiti, hearths and all kinds of destructive actions, someengravings correspond to a very simple figure of bison with one horn connected to the forehead and the other to the hump (D.I.1), to other two bison heads (D.I.2, D.III.1), two horns of bison (D.III.2) and to an another indeterminate animal head (D.II.1), and to non-figurative digital tracings (D.IV.1).

### Aitzbitarte IX

**Fig 12:**
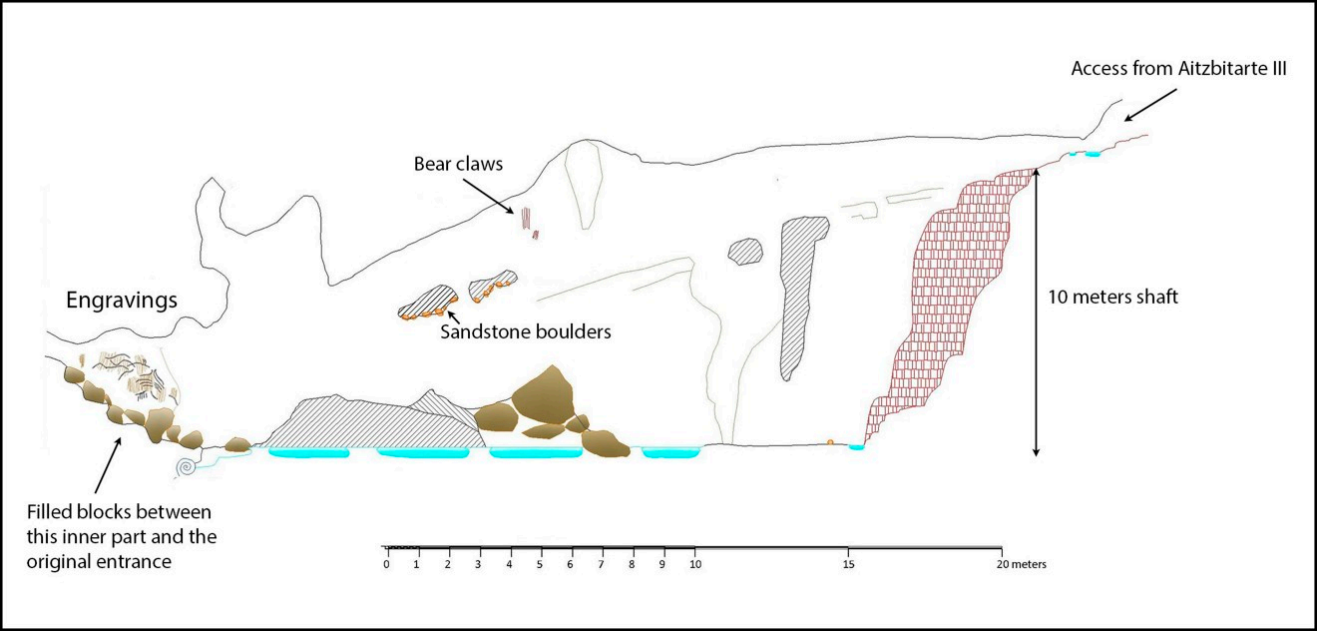
Plan of Aitzbitarte IX. The decorated area is indicated (J. Busselo).

**Fig 13:**
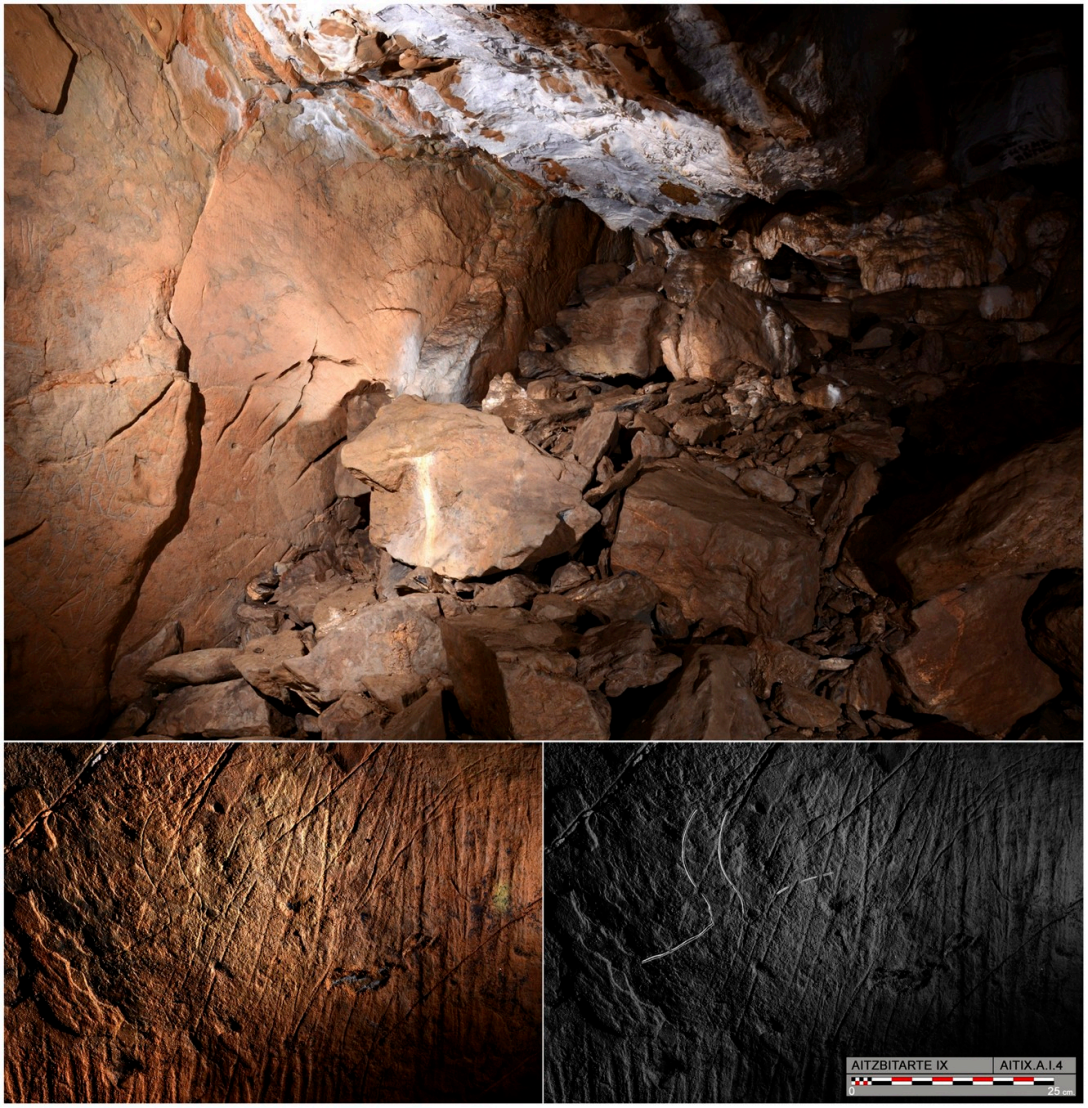
The engravings from Aitzbitarte IX. Top: Collapsed passage in Aitzbitarte IX with the decorated panel on the left. Bottom: Photograph and tracing of the engraved bison head A.I.4 in Aitzbitarte IX (O. Rivero and D. Garate).

The original entrance of this cave is now blocked by the partial collapse of the roof, which affects the first part of passage. Current access is down a 10m shaft that connects the end of Cave III (upper level of the system) with the bottom of Cave IX (lower level of the system: Fig 12). Turning back in the cave over a few metres to the inner side of the collapse, on what would be the right-hand wall coming from the entrance, a flat surface about 2m long is decorated with engraved motifs (Fig 13 top). It is covered with vertical lines more or less in series that form wide but shallow grooves. Among them, some finely-engraved lines can be observed and among them a triangular shape and the head and back of a bison (A.I.4) with horns in a single plane; one connected to the forehead and separate, the other prolonged in the animal’s back (Fig 13 bottom).

The position of the decorated panel, 3m above the original floor but only 1m above the collapsed boulders, suggests that part or all the collapse would have occurred before the motifs were engraved. Therefore, speleothems of both parts of the collapse (Fig 4) have been dated to determine the state of this passage in the time when the cave was decorated in the Palaeolithic. Three speleothem fragments (AIT_IX-1, AIT_IX-2 and AIT_IX-3) extracted from between the boulders in the upper part of the collapse reached from the entrance of Aitzbitarte IX were analysed and the first two have ages of 17.657 ± 220 and 125.712 ± 1333 ky respectively (Table 1). Therefore, the collapse must have startedbefore those dates. Speleothems *in situ* on top of the collapsed boulders in the decorated chamber have also been dated (AIT_IX-4ext, AIT_IX-4int and AIT_IX-5). They correspond to the Holocene and therefore cannot justify that the last boulder collapses took place before the human occupation. In sum, the results of the U/Th dating suggest that the Palaeolithic access was via the shaft at the end of Cave III (S1 File).

**Table 1:** Cristal fabric (based on Frisia, 2015) and results of the U/Th dating of speleothem samples from Aitzbitarte IX. Dating performed in CENIEH Uranium Series Laboratory (Spain).

Sample	AIT_IX-1	AIT_IX-2	AIT_IX-3	AIT_IX-4ext	AIT_IX-4int	AIT_IX-5
Crystal fabric	Columnar porous	Columnar open	Dentdritic porous	Columnar open	Columnar open	Columnar
**U (μg/g)**	**0.033 ± 0.002**	**0.020 ± 0,001**	**0.061 ± 0.002**	**0.045 ± 0.002**	**0.026 ± 0.001**	**0.027 ± 0.001**
**Th (μg/g)**	**0.026 ± 0.001**	**0.087 ± 0,004**	**1.022 ± 0.051**	**0.005 ± 0.0003**	**0.003 ± 0.0001**	**0.011 ± 0.001**
**230Th/232Th x10-6**	**4.4 ± 0.001**	**4.0 ± 0,001**	**3.9 ± 0.002**	**11.1 ± 0.02**	**9.6 ± 0.02**	**4.5 ± 0.01**
**δ234U**	**384 ± 3**	**511 ± 5**	**412 ± 3**	**1431 ± 6**	**714 ± 8**	**678 ± 6**
**230Th/238U**	**0.210 ± 0.002**	**1.092 ± 0,002**	**3.944 ± 0.004**	**0.074 ± 0.017**	**0.062 ± 0.016**	**0.111 ± 0.007**
**Age 230Th (BP)**	**17 657 ± 220**	**125 712 ± 1333**	**-**	**3238 ± 749**	**3931 ± 1060**	**7288 ± 355**

### Gravettian rock art in Aitzbitarte caves

The three decorated caves in Aitzbitarte Hill described above display strong similarities looking to the thematics, the technical procedures and formal conventions employed, and in the topographic position of the rock art.

Without forgetting possible preservation biases, which may have erased completely some representations and that have affected others precluding a clear identification of the engraved motifs, it can be said that a balance exists between the figurative (animals) and non-figurative depictions (signs and lines) (Table 2). Among the former, bison, determined by outstanding humps, are the most frequent animals in all three caves and the only one in Aitzbitarte V and IX. In contrast, in Aitzbitarte III they are accompanied by horses and aurochs (determined by pointed and sloping horns, absence of hump and massive body) in smaller numbers. Interestingly, bovids were the most consumed animal in the levels of the Gravettian occupation in Aitzbitarte III, especially in the Middle Gravettian levels in the interior excavation area [35, 36]. A single depiction of a bird has been identified. Among the non-figurative motifs there are no conventional signs.

**Table 2:** Graphic units in the Gravettian representations in Aitzbitarte Caves III, V and IX.

Cave	Bison	Horse	Aurochs	Ibex	Bird	Animal	Sign	Lines	Total
**III**	11	5	2	1	1	1	0	15	**36**
**V**	7	0	0	0	0	1	1	1	**10**
**IX**	1	0	0	0	0	0	1	7	**9**
**Total**	19	5	2	1	1	2	2	23	**55**

All the representations in the three caves were engraved. Incisions with a V-shaped cross-section have been observed in all the graphic units except in the case of the indeterminate animal B.VII.1 from Aitzbitarte III and the series of vertical lines A.I.1 in Aitzbitarte IX, which were engraved with a blunt implement. The lines were drawn with a single action, and no evidence has been observed of deepening the same line or the juxtaposition of several incisions by several movements of the implement. In this way, the outlines of the figures were created by a single line as the result of a single incision with the tool.

From the point of view of their representation, most of the animals are partial figures as only four of the figures are complete, and they are limited to the fore-quarters of the animals. Except in the case of one bison, they are characterised by incorrect perspective, in which upper features like the horns and ears and the lower limbs are depicted as frontal views. There are few anatomical details, only the eyes in the two horses in Aitzbitarte III and the hooves of the aurochs A.III.4 in the same cave. A specific artistic convention seen in most of the bison consists of representing the horns separate from one another; one connected to the frontal-nasal line and the other to the hump.

Finally, the graphic units are not distributed at random in the three caves. They are all located in secondary areas, away from the main passages or routes through the cave, in places with low visibility. Access is quite complicated in all cases, either down a narrow descending crawl in Aitzbitarte III or by climbing in Sector A in Aitzbitarte V. The geomorphological reconstruction of Aitzbitarte IX is not easy but the dates obtained for speleothems show that the collapse had largely occurred before the decoration, and therefore access must have been down a 10m shaft at the end of Aitzbitarte III (S2 File).

### Inside Gravettian art and societies

The animal depictions found in Aitzbitarte III, V and IX are framed within a graphic tradition (subject matter, artistic conventions, associations, etc.) attributed to the Gravettian cultural complex [47, 51]. One frequently recurring aspect is the incorrect perspective of the figurative depictions, which is seen especially in the horns [12] and in limbs that often take the shape of a ‘double Y’ [52]. These specific features are absent during the Magdalenian [53]. In fact, this incorrect perspective manifests itself differently in the pre-Magdalenian graphic traditions; in Cantabrian Region the deeply engraved animal in the outside of some caves or rock-shelters only represent one leg per pair (e.g. La Lluera, Chufín, Hornos de la Peña, Venta la Perra, etc.); this is the same case for open-air rock sites as Foz Côa, Siega Verde, Domingo García or also for some caves around Western Europe [54]. Also, in Cantabrian Region, red dotted animal paintings (e.g. La Pasiega, El Pendo, Covalanas, Arenaza, etc.) show two legs per pair but the front ones are represented without perspective and the back ones in two planes with very specific exceptions [46]. The systematic representation of both legs by pair and without perspective is a characteristic of the caves on which we based our study, even if it can appear marginally in other sites where other solutions are preferential [52], or in post-Magdalenian chronologies [55, 56]. This is to say, we can identify different formal solutions when representing both the lower and upper extremities, and compare them between caves.

#### Formal concepts in animal symbolism

To establish these artistic links more robustly, these conventions related to the use of the perspective have been defined precisely. Then all the documentation in the European archaeological record -including Aitzbitarte caves- has been examined to identify these specific similarities in other portable and parietal ensembles, using some basic morphotypes. This work has considered recent finds in Alkerdi 2 [57], the decorated pebble from El Pendo [58] and other ensembles that have not been previously considered (Abri Laraux, Abri Labattut, Abri Pataud, Erberua and Pergouset). It also includes the portable and parietal ensemble at Parpalló Cave [59], as several motifs in that cave display the same conventional features, as well as a figure in La Pileta Cave [60] and two figures of El Moro [61]. As we have pointed previously, there are also some marginal sites or depictions in where these formalisms can be found, but where other conventionalism abound, like Mazouco [62], El Niño [63], La Griega [64], La Clotilde [65] and El Rincón [52]. This is to say, in this study we have considered for the first time, the sites and the animal figures where we can find the conventions related to the perspective, in order to define their geographical distribution and specifics.

#### Analysis of the conventions identified

In the case of the engraved figures in Aitzbitarte Caves III, V and IX, a series of conventions are linked with the way the bovid (bos/bison) horns are connected, and with the fore and hind limbs of the herbivores. Both criteria have been used to enable the identification of parallels in parietal and/or portable depictions at other sites. This is to say, we have limited our study to some easily identifiable conventions, refusing to include those whose recognition may be more ambiguous in order to avoid erroneous results. These formal criteria have been defined as attributes and values (Table 3) in order to perform Correspondence Factorial Analysis (CFA) and study the relationship between the animals represented, graphic conventions and their geographic distribution.

**Table 3:** Attributes and values used in the study.

Attributes	Values	Code
Motif	Bison	bs
	Aurochs	au
	Horse	hrs
	Cervid	cer
	Ibex	ibx
	Uncommon	inf
	Unidentified	ind
Horn convention	Missing	msh
	Continuous	cnh
	Strokes	str
	No convention horn	nch
	No horn	nho
Front leg convention	Missing	msf
	Rectangular	ref
	Pointed	pof
	Round	rof
	Other	otf
	No convention front	ncf
	No front leg	nfl
Back leg convention	Missing	msb
	Rectangular	reb
	Pointed	pob
	Round	rob
	Other	otb
	No convention back	ncb
	No back leg	nbl
Region	Western Pyrenees	WPR
	Central Pyrenees	CPR
	Quercy	QUE
	Périgord	PER
	Mediterranean	MED

**Fig 14:**
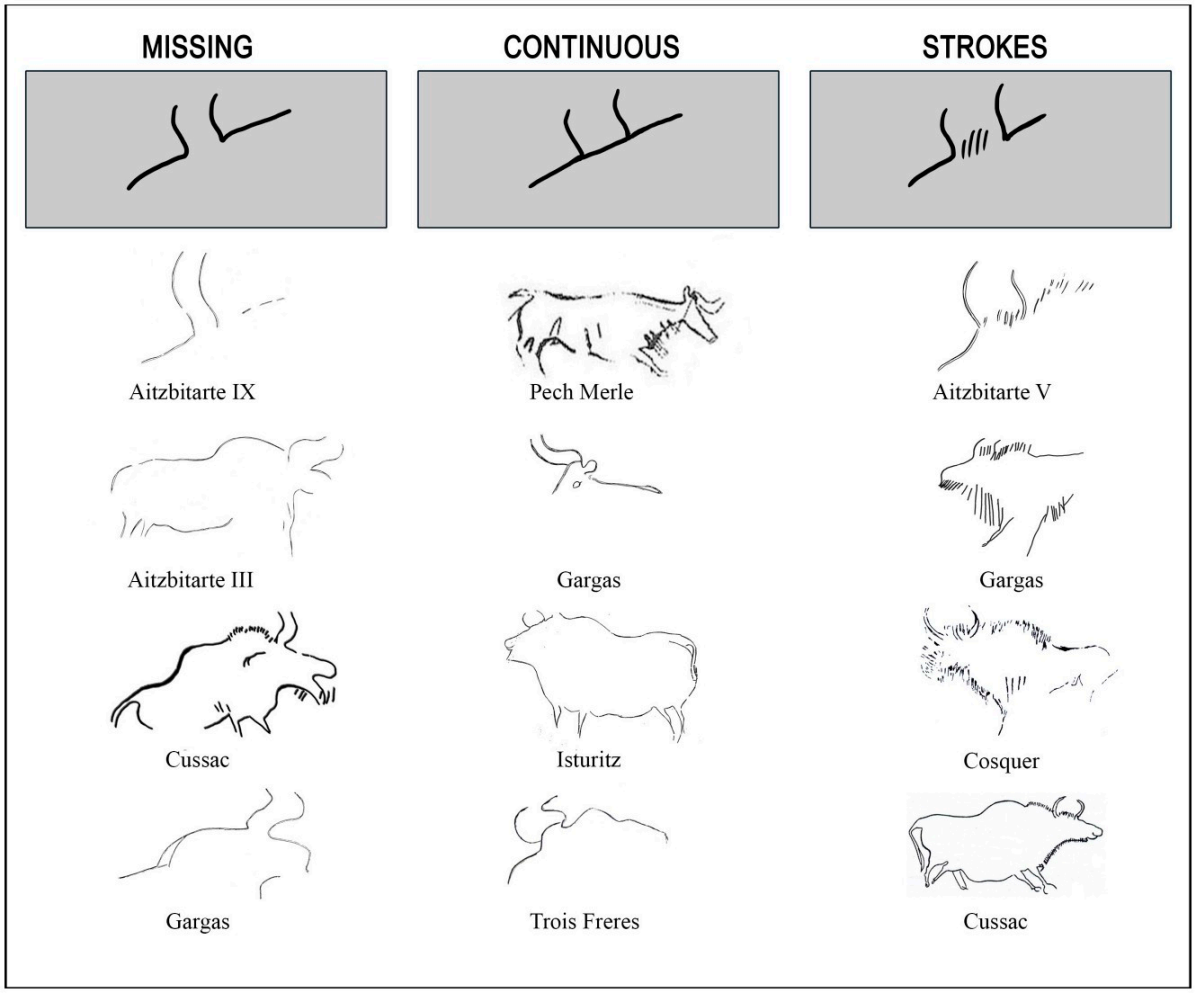
Different conventions to represent the space between the horns in bovids: Aitzbitarte V, IX, Isturitz [15], Cosquer [14], Gargas [12], Trois Frères [66], Cussac [13].

In the first place, one of the most representative conventions in this type of figure is the way of depicting the space between the horns without perspective of bison or aurochs. In the analysed figures, the space may be absent, represented by a continuous line, or by a series of short lines or strokes (Fig 14). The horns are depicted in most cases as an appendix or either the forehead or the hump, leaving the space between them empty of filling it with a series of lines, as seen in the Aitzbitarte caves. In these cases, the perspective can vary from absolute profile to double, frontal or semi-twisted profile. We have revised all the Gravettian/Solutrean sites in order to find these conventions, considering only those where they are identifiable in Western Europe.

A total of 97 representations of bovids (70 bison and 27 aurochs) in which one of the three types of convention can be observed have definitely been identified in the sample (S1 Table). The distribution of depictions among the sites considered is: Abri Pataud: 1; Aitzbitarte III: 2; Aitzbitarte V: 2; Aitzbitarte IX: 1; Alkerdi 2: 1; Cosquer: 11; Cussac: 15; Erberua: 2; Gargas: 32; Isturitz: 11; Parpalló: 1; Pech Merle: 6; Pergouset. 2; Rocadour: 3; Trois-Frères: 7. Despite the small number of individuals, factorial analysis is able to study the variability in these types of conventionalisms, in association with one or other bovid species or a particular geographic area.

The analysis was performed with a Burt table with 11 x 11 attributes and values. Together with the formal criteria described above, it has included the criterion of area, differentiating between the regions traditionally defined in Palaeolithic art studies: the western Pyrenees (we have included here the new discoveries), the central Pyrenees, Périgord, Quercy and the Mediterranean.

**Fig 15:**
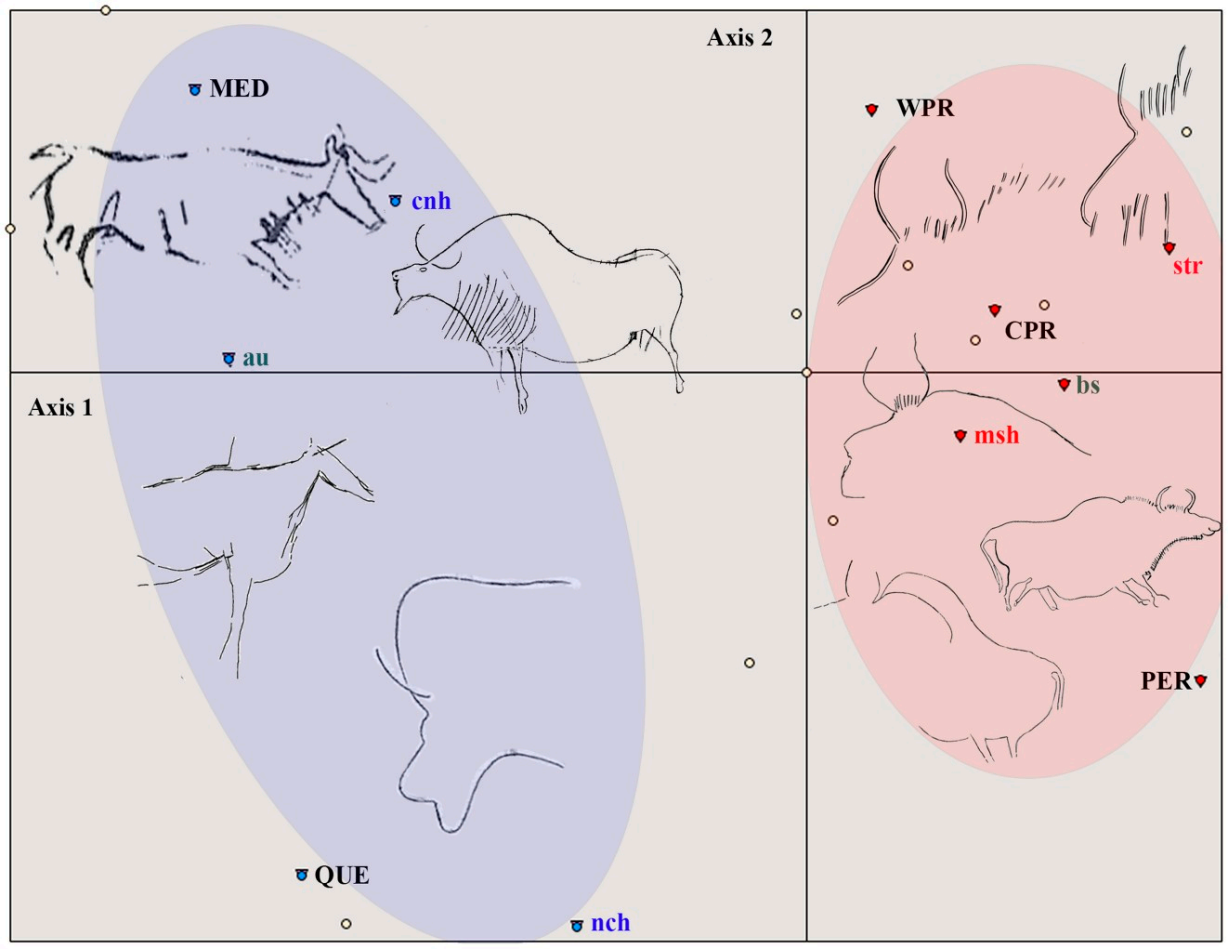
FCA multivariate plot of the studied corpus of 11x11 criteria in the main factorial plane [1,2]. The two-colored groups are generated by ascending hierarchical clustering (MED: Mediterranean, QUE: Quercy, PER: Perigord, CPR: Central Pyrenees, WPR: Western Pyrenees).

**Fig 16:**
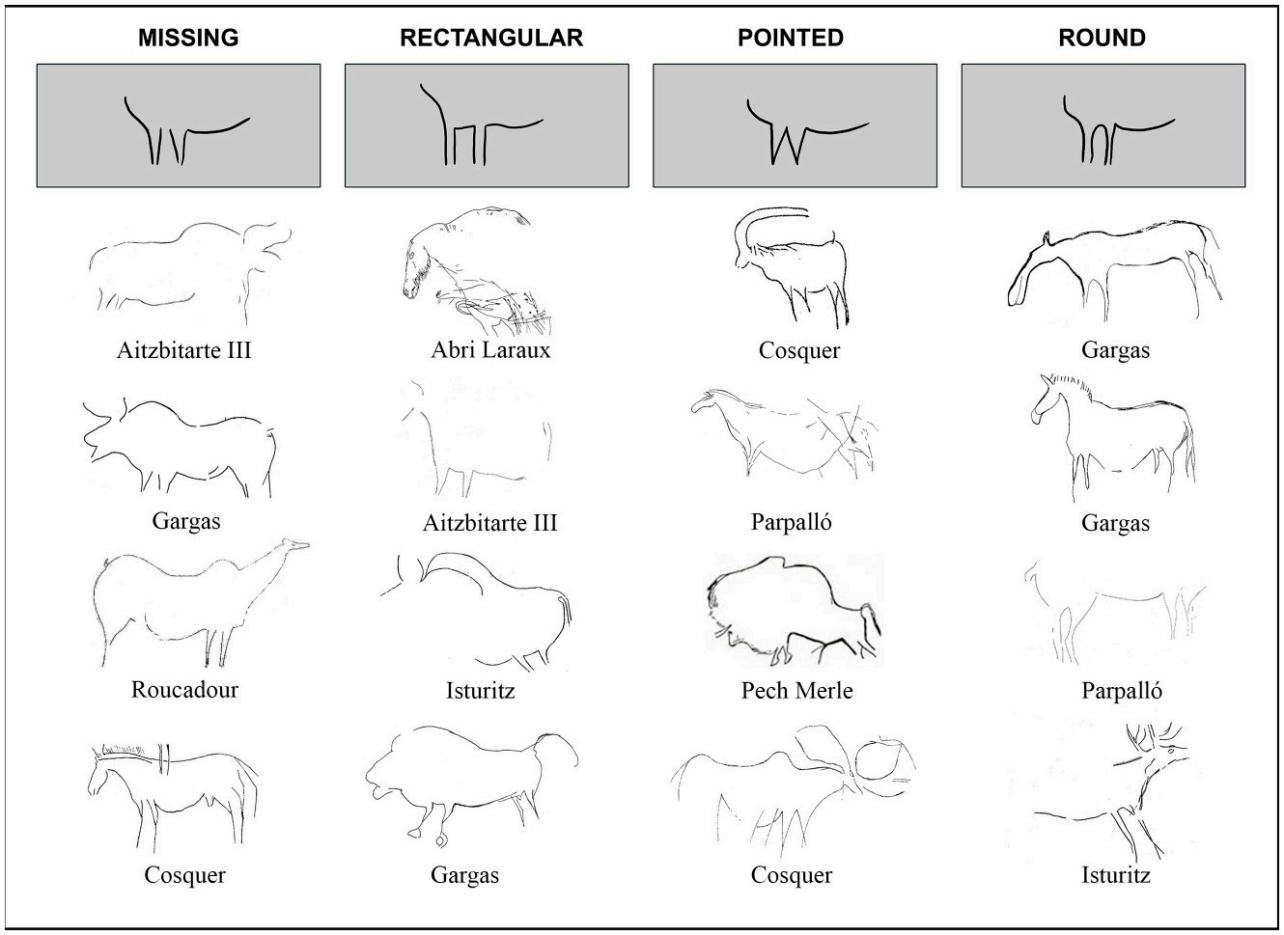
Different conventions to represent the connection between the two fore- or hind-limbs of herbivores: Aitzbitarte III [2], Isturitz [15], Cosquer [14], Gargas [12], Abri Laraux [67], Pech Merle [68], Parpalló [69], Roucadour [70].

The distribution over the factorial plane [1,2] shows two groups differentiated on either side of Axis 2 (Fig 15), established according to Ascending Hierarchical Clustering (AHC). In these groups we see that the western Pyrenees is situated on the right-hand side of the plane, together with the central Pyrenees and Périgord, and these three areas are articulated around bison figures and the ‘stroke’ convention. On the left-hand side, Quercy and the Mediterranean are associated with aurochs and the ‘continuous’ convention and the absence of conventions (Fig 16).

Although the number of individuals does not allow statistically significant correlations between the different attributes (we only find as characteristics of the blue class, with a probability of above 95%, the criteria ‘stroke’, ‘missing’, ‘bison’), this analysis reveals a trend that, as will be seen, is confirmed by the analysis based on the limb graphic conventions. They indicate regional differences in the way of representing particular animals with specific formal characteristics.

The formal variability in the representation of herbivore limbs when they are depicted in a frontal perspective has also been analysed. Several ways exist to depict the union between the two limbs, either fore- or hind-legs. This may be missing, rectangular, pointed or rounded. Again, we have revised all the Gravettian/Solutrean sites in order to find these conventions, considering only those where they are identifiable in Western Europe.

Out of the figures that have been studied and clearly individualised with the available information, a total of 252 figures have been selected in which at least one of the four ways to depict the limbs can be identified. These contain a total of 308 samples of fore- and hind-limbs. The distribution of depictions among the sites considered is: Abri Labattut: 2; Abri Laraux: 1; Aitzbitarte III: 2; Aitzbitarte V: 2; Alkerdi 2: 1; Brassempouy: 2; Cosquer: 75; Cussac: 22; El Pendo: 1; Gargas: 60; Isturitz: 23; Le Portel: 2; Parpalló: 22; Pech-Merle: 12; Pergouset: 3; Rocadour: 13; Trois-Frères: 9.

The FCA has been performed with a Burt table using 26 x 26 criteria (S2 Table). As before, the areas where the sites are located have been codified. The criteria ‘other front’ and ‘other back’ and the regions have been emplaced as Supplementary Elements so that they do not participate in the distribution of the factorial plane.

**Fig 17:**
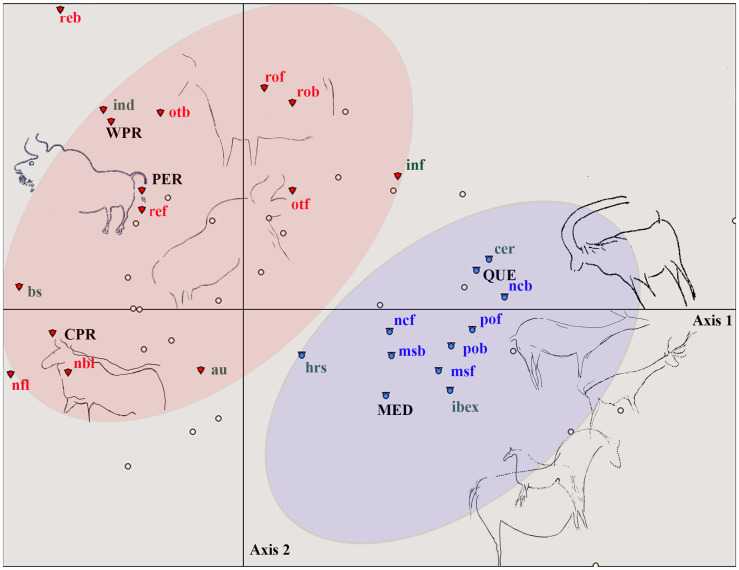
FCA multivariate plot of the studied corpus of 26x26 criteria in the main factorial plane [1,2]. The two-coloured groups are generated by ascending hierarchical clustering (MED: Mediterranean, QUE: Quercy, PER: Perigord, CPR: Central Pyrenees, WPR: Western Pyrenees).

The outcome of the analysis, combined with AHC, shows two groups clearly differentiated on each side of Axis 2. As in the previous analysis, these groups are the western Pyrenees, central Pyrenees and Périgord on one side and Quercy and the Mediterranean on the other side (Fig 17).

The red group, to which the western and central Pyrenees and Périgord belong, is characterised with a probability of above 95% by the attributes *reb* (rectangular back leg), *ref* (rectangular front leg), bison and the Pyrenean areas and Périgord.

The blue group is characterised by the criteria ‘missing’ in front and back legs, and by the criteria ‘pointed’ in back and front legs, as well as by ‘cervids’ and ‘ibex’. These criteria are associated, with 95% probability, with the Mediterranean and Quercy.

If the Z-Score test is added to this analysis (Table 4) and applied to the same criteria to determine their degree of correlation, several significant correlations of above 90% probability are found.

**Table 4:** Z-test. Probability that the co-occurrence of two attributes is not due to the fluctuations of sampling. Positive values = excess with respect to a random distribution; negative values = default with respect to a random distribution. The percentages indicate the probability that the co-occurrence between the two variables is not due to fluctuations in the sample.

Attributes	Ibex	Bison	Horse	Pointed	Rectangular
**Mediterranean**	98%	*- 99*,*9%*		91%	*-98%*
**Central Pyrenees**			*-96%*	*-98%*	
**Périgord**	*-94%*				
**Western Pyrenees**	*-95%*				95%

The results of both analytical tests show that both the subject matter and the conventions employed in the representation of the limbs differ from one region to another.

#### Chronological attribution

The chronological information available for some of the sites (S3a and S3b Table) repeatedly points to a time within the limits of the Gravettian complex, but some of them are younger coinciding with the Solutrean. Direct dates are available for parietal art in the caves of Cosquer [71] and Pech Merle [72], for the context of the art in the case of bones wedged in the wall at Gargas [73, 74], for burials at Cussac [13] and for the stratigraphy in which portable art was found at Parpalló [69], Abri Laraux [67], Abri Pataud [75], Abri Labattut [76], Isturitz [15] and Gargas [77].

The radiocarbon determinations obtained directly for black paintings at Cosquer are hard to interpret. In the case of Horses 1 and 5 in Sector 101, the new dates provide certain coherence as the former is dated to 30.1–29 ky cal BP and the latter to 29.4–28.1 ky cal BP in the case of the oldest result. This is very similar to the result of the humic fraction sample of the other three dates for the same figure. The other horses that have been dated, numbers 7, 17 and 57, a *megaloceros* and a feline provided much later results corresponding to the Solutrean. These could explain the more recent dates for Horse 5 as due to the action of repainting the figure. In the case of Bison 1 and 2, they have both been dated three times with very coherent results individually (for the former of 22.8–21.3 ky cal BP and for the latter of 32.4–29.6 ky cal BP) but thus differ by about 10,000 years in age despite their great formal similarity. Indeed, the 41 dates obtained for paintings and other archaeological remains in Cosquer indicate that the cave was decorated in three different phases during this period [71].

In the *Grand Salle* of Isturitz Cave two levels, III and IV, have been attributed, with reserves, to the Gravettian [78]. While no radiometric dates have been published for this sector, in the *Salle des Phosphates* a result of 31.3–30.7 ky cal BP has been obtained for a bone fragment recovered from the stratigraphic section and this could correspond to one of the two levels documented in the contiguous chamber. The ephemeral archaeological context associated with the parietal art in Alkerdi 2 cave [57], which is still in the course of being studied, provided a practically identical chronology.

In the case of Gargas, two dates of about 31.7–29.9 ky cal BP are available for bones wedged in the wall [73, 74], associated spatially with the hand stencils. Portable art displaying the same conventions as described in the present study was documented in a stratigraphic level more recent than 30.7–28.5 ky cal BP [77], which may indicate at least two different phases of decoration in the cave: one for the hand stencils and a later one for the portable and parietal engravings. On the contrary, in the case of Roucadour, the analysis of the overlays indicates that the hand stencils are interspersed between engraved animal phases -represented with the studied conventions- in panel I or superimposed on them in panel XII [79].

Dates for the archaeological context in Cussac Cave document human activity in about 29.7–28.7 ky cal BP [80], while the dotted horses in Pech Merle have been dated directly to 29.5–27.8 ky cal BP [72]. In the case of the portable art in the rock-shelters of Pataud [81] and Laraux [82], the stratigraphic sequences with portable art objects correspond to a more recent chronology, but according to determinations with very large standard deviations as they were obtained about fifty years ago.

In the central Mediterranean, some researchers have stated that “it is practically impossible to establish differences between Gravettian art and early Solutrean art, at least in the portable objects from Parpalló” [83]. This may be a case of persistence of the graphic tradition during the Solutrean. Indeed, it is possible to observe similarities in the Solutrean both at Parpalló and proximate sites (Mallaetes and Les Meravelles) [59] and in the south of the Iberian Peninsula [60]. The way of representing hinds’ ears with the ‘trilinear convention’ during the Solutrean in southern Iberia [84] is very similar to the conventionalism by which the space between the bison horns is absent in the Gravettian. The same may be true of Cosquer Cave [71], where one of the three decorative phases identified in the cave is attributed to the Solutrean despite the maintenance of Gravettian stylistic conventions. This is to say, chronological data is indicating a continuity of some artistic traditions throughout Gravettian and Solutrean periods, at least in the Mediterranean area.

In brief, the contextual or stratigraphic dates that points to older chronologies (early-middle Gravettian) are those obtained in the S/W of France, which would be the closer to Aitzbitarte. Similar early dates have been obtained in the excavations of Aitzbitarte III entrance (Units IV-Va) [6, 28], which is located just beside the decorated sector. As it extends to the Mediterranean area, more recent dates begin to appear, reaching the Solutrean phases (both in their initial and final stages) in the more remote areas (Cosquer and Iberian Mediterranean coast). In fact, the prolonged sequences, and the probable persistence of conventionalisms observed in Parpalló and Cosquer, seem to point in this direction. Something similar was proposed for the central area of France, in which "aviform" signs, typical of Gravettian phases, could have persisted during Solutrean in Placard cave [17].

#### Geographic distribution

**Fig 18:**
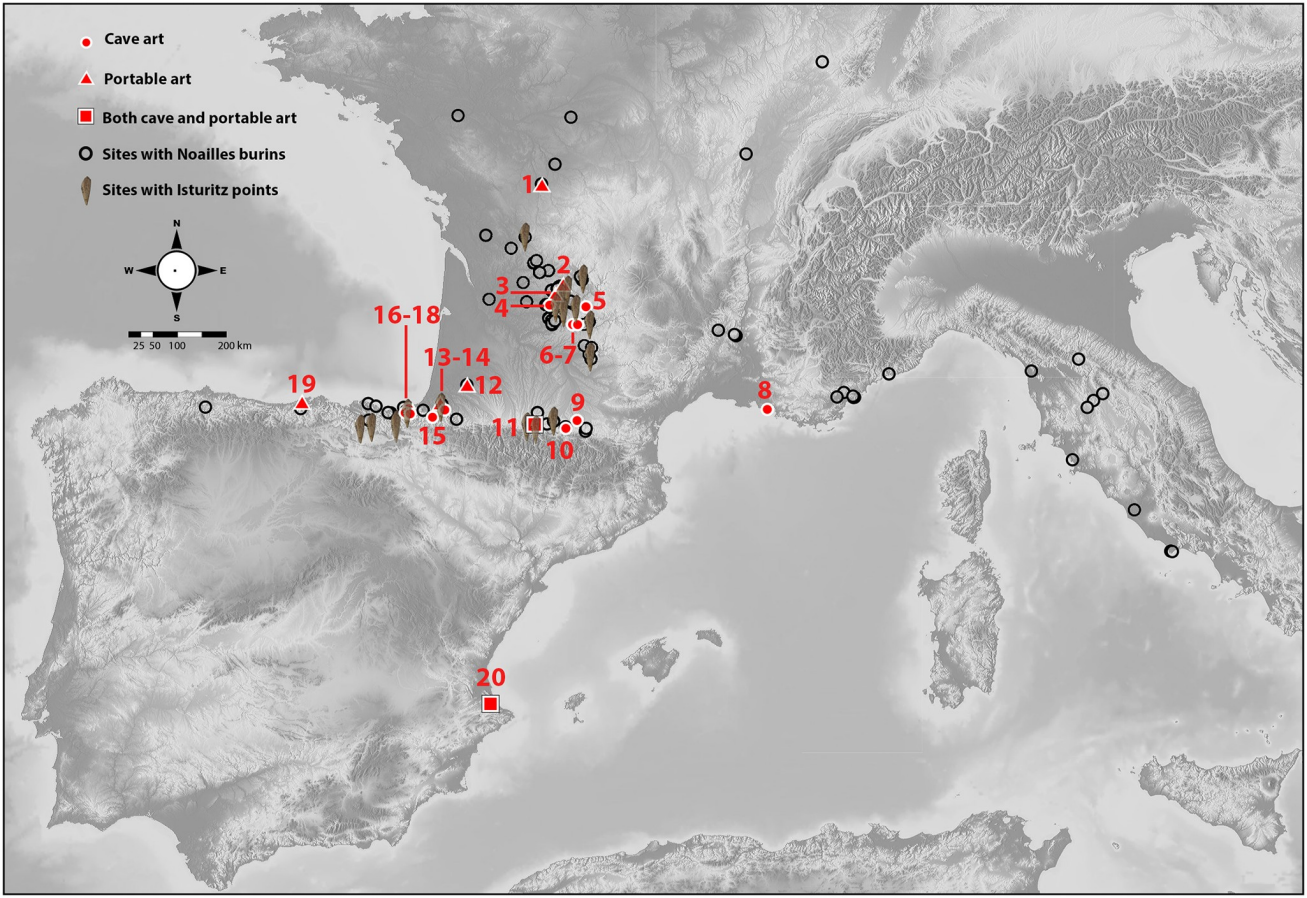
Sites with graphic units in parietal ensembles (circle), on portable objects (triangle) or both (square) (base map: https://maps-for-free.com): 1. Abri Laraux; 2. Abri Labattut; 3. Abri Pataud; 4 Cussac; 5. Roucadour; 6. Pergouset; 7. Pech Merle; 8. Cosquer; 9. Le Portel; 10. Trois-Frères; 11. Gargas; 12. Brassempouy; 13. Erberua; 14. Isturitz; 15. Alkerdi 2; 16. Aitzbitarte V; 17. Aitzbitarte III; 18. Aitzbitarte IX; 19. El Pendo; 20. Parpalló. Sites with Noailles burins (black circle). Sites with Isturitz-type points (icon of a point) (I. Intxaurbe).

These graphic conventions have been identified in about twenty parietal and/or portable ensembles, especially in connection with the depiction of some features (mainly horns) and the limbs (Fig 18). As we have already mentioned, they can also appear sparingly in sites where other perspective solutions are preferential (red dotted animal in cave or picketed animal in open-air) or in caves without a defined style (e.g. Clotilde or Rincón in Cantabrian Region).

The analysis has revealed formal unity between sites located over a very wide area. The main concentrations are in two nuclei: the Western Pyrenees in the area of influence of Isturitz, where no evidence was known a few years ago (Brassempouy, Erberua, Isturitz, Alkerdi 2 and Aitzbitarte III-V-IX), and in Périgord and Quercy (Abri Labattut, Abri Pataud, Cussac, Roucadour, Pergouset and Pech Merle). Other especially profuse ensembles are found in Gargas, in the central Pyrenees; Cosquer, next to the mouth of the River Rhone; and Parpalló, on the central Mediterranean coast in the Iberian Peninsula. Therefore, the distribution area is much larger and slightly different from how it has been conceived in the past, when it was limited to Gargas, Cosquer and the caves of Cussac, Pech Merle and Rocaudour in Périgord [16].

#### Mobility and contacts between social groups

The aforementioned distribution of sites suggests a concentration of these artistic conventions in two main areas, the Périgord and the Western Pyrenees. Interestingly these two areas are the main distribution area of Isturitz type points [33] and the Noaillian Gravettian (see distribution map in [85]). In the Cantabrian Region the western limit of the Noaillian Gravettian can be established in the River Nervión, with Bolinkoba, Antoliñako Koba and Atxurra as the westernmost representatives of this cultural complex. Nevertheless, some Noailles burins can be found in Cantabria or even Asturias, but always in small numbers except at La Viña [86], isolated for the moment in the Western Cantabrian region. This is also exactly the distribution area of Isturitz points. As we have discussed in a previous paper, not a single piece to the west of Bolinkoba can be confidently interpreted as an Isturitz point [33]. Therefore, Aitzbitarte is situated in the middle of one of these major areas (Périgord and Western Pyrenees), in a clear relationship with Isturitz, a site who’s Gravettian strongly resembles the one in Aitzbitarte III interior Unit VI-V. The unity of this area has also been documented through the circulation of raw materials (see [87], as flint from Chalosse, Bidart, Urbasa, Treviño and Barrika is common in both sites. This circulation of raw material also links this area with the central Pyrenees, more specifically Gargas [88], a major Noaillian site, and these two areas (western and central Pyrenees) with the Dordogne [89]. Furthermore, in this Western Pyrenees area, most of the Gravettian sites can also be attributed to the Noaillian facies. This is the case of Antoliñako Koba, Bolinkoba, Atxurra, Amalda, Ametzagaina, Aitzbitarte III, Isturitz, Gatzarria and Brassempouy [31, 32, 90–98].

However, there are some notable exceptions such as Alkerdi in the Pyrenees or the open-air site of Mugarduia South, where only few atypical Noailles burins have been recovered [99–101]. There are also references to a Gravettian without Noailles burins in Aldatxarren, but it has never been published in detail [102]. This means that the Gravettian in the area around Aitzbitarte, where this rock art style appears, can be basically attributed to the Noaillian facies, from the very beginning (see the early dates for Amalda and Aitzbitarte III; [6]) to the end of the regional Gravettian (see the dates for Aitzbitarte III-exterior Unit III; [28]. Also, considering the early dates of the Gravettian in Aitzbitarte III and Amalda [6], it has been considered a possible source area for this technocomplex in Western Europe, probably as a result of technical transformations in the Late Aurignacian [8].

The distribution of the Early Noaillian seems to be circumscribed to the Pyrenean Region [103], and later on it expanded to other regions of Western France (Périgord and Quercy), and even further towards Eastern France (Provence) and north-western Italy (Liguria). In these regions, the Noaillian is more recent, closely adjusting to the concept of middle Gravettian.

As we have observed, assigning a specific chronology to this graphic tradition is difficult, and this is something that can be extended to the whole of pre-Magdalenian art. This has to lead us to consider the possibility that different well-defined graphic traditions could coexist within the same geographical and chronological framework, as seems to be the case on the Cantabrian coast [46]. The graphic style present in Aitzbitarte, until now was exclusive to the South of France, in addition to the Mediterranean coast. With this discovery, along with the decorated pebble of El Pendo [58] (Rivero *et al*., submitted), this graphic tradition also seems to be present on the Northern Iberian Peninsula, albeit occasionally, and perhaps in coexistence with other graphic families (such as red dotted animals) stylistically dated trough portable art with similar chronologies [104]. This in turn could allow exchange networks, and specific assumptions of certain conventions, as the presence of identified conventions in marginal cases and the repetitions of certain recurrences in different graphic families seem to demonstrate (e.g. *bec du canard* and *step crin* convection in horses, among others) [19].

## Conclusions

The Gravettian cultural complex is characterised in symbolic terms by the human burials and very specific graphic elements, particularly the famous ‘venuses’ or female figures, and stencilled hands, which are found across much of central-western Europe [11]. In fact, Gravettian is considered the first pan-European culture during the Upper Palaeolithic [105] where several specifies has been recognized around the whole territory -facies- [7], even if this concept has been recently questioned [106].

The discovery and study of Palaeolithic parietal art in three caves in Aitzbitarte Hill (northern Spain) have contributed new information about the distribution of some characteristic Gravettian graphic conventions as their presence in the northern Iberian Peninsula has thus been confirmed. Additionally, the exhaustive review of the parietal and portable artistic record, together with chronological data, has enabled a new definition of the geographic distribution of ensembles displaying those conventions. It has been found to cover a much larger geographic area than previously thought (Central Pyrenees, Périgord and the French Mediterranean).

In fact, this new size of the distribution area of the artistic conventions identified in Aitzbitarte III, V and IX, which characterize a specific Gravettian art within Western Europe, corresponds to the distribution of the well-known Isturitz-type points. Interestingly the distribution area of the Noaillian facies goes further than the distribution area of these conventions, given that there are no examples of this art in Italy, where the facies is significantly more recent. Moreover, the distribution area of these conventions expands towards the Mediterranean coast of the Iberian Peninsula (Parpalló) in later periods, in the Solutrean, although no site has ever been described as Noaillian in that region [107, 108].

The statistical analyses of the graphic conventions show a close relation between Périgord sites (especially Cussac) and the Western and Central Pyrenean sites, while the Quercy caves are more closely related to the Mediterranean side (Cosquer). The link between Quercy and Mediterranean cost have been underlined before by other prehistorians [109, 110], considering different graphical conventions. This can be explained by two slightly graphical differences and/or by a chronological change in these analysed convention priorities, within the framework of a more diverse artistic tradition that falls between the Gravettian and the Solutrean [47]. In fact, other clues, like flint procurement, the engraved ribs, incised ivory pieces or Atlantic marine resources, reinforce the contacts between the Central and Western Pyrenees and the Périgord [88, 111]. The scarce resolution of the dating record does not allow us to propose a hypothesis to explain these regional differences but a long persistence of these conventions is suggested by the dates. This is to say, we have an early Gravettian context for Aitzbitarte III, Isturitz and Alkerdi 2 in the Western Pyrenees, a middle Gravettian context for Gargas related with portable art and an even earlier one for the wedged bones in the walls related to hand stencils, and also a middle Gravettian age at Cussac for the archaeological context which is also close to the single direct date for Pech Merle’s dotted horses. The only direct dates for these kinds of images come from Cosquer cave, where at least three phases of decoration are discriminated by radiocarbon dating within a 10ky range; from the Early Gravettian up to the Solutrean. Stratigraphical information related to the portable art at Abri Laraux and Pataud seems to indicate a more recent Gravettian and in the case of Parpalló, the pieces were found in Solutrean levels.

In conclusion, specific graphic unity in both portable and rock art is mainly focused on the Western and Central Pyrenees and Périgord, coinciding with other traits–Isturitz points and flint provenance, for example- of the Noaillian facies in the Middle Gravettian. This reflects an intense network of social interaction for both technological and symbolical elements. In the latter case, a slightly formal difference with the Quercy region can be observed, even though it is close to Périgord and the chronology seems similar in some cases. It seems much more difficult to understand the case of Cosquer Cave, with its three phases of decoration employing the same conventionalisms during 10,000 years and nothing similar in the same region or in the neighbouring Ardèche. This phenomenon has no parallels among the other caves, but we know that these conventions persisted on the Iberian Mediterranean coast during the Solutrean. This is to say, even if this art seems to start at the Noaillian sites in the Western Pyrenees, where the oldest sites related to this technocomplex are found (Aitzbitarte III, Atxurra, etc.) and then expands to the Central Pyrenees and to the Périgord, a reminiscence later affected a wider geographic distribution.

## Supporting information

S1 Table (DOCX)

S2 Table (DOCX)

S3 Table (DOCX)

S1 File (DOCX)

S2 File (DOCX)
