# Supplementary material for: Redefining shared symbolic networks during the Gravettian in Western Europe: New data from the rock art findings in Aitzbitarte caves (Northern Spain)
Source: PLoS One. 2020 Oct 28;15(10):e0240481. doi: 10.1371/journal.pone.0240481 (PMC7592797; doi:10.1371/journal.pone.0240481)
Supplement: S1 Table — (DOCX) [file pone.0240481.s001.docx]

| **Code** | **Site** | **Region** | **Motif** | **Convention**  **Horn** | **References** |
| --- | --- | --- | --- | --- | --- |
| AP1 | Abri Pataud | Perigord | Bison | mch | Movius, 1977: p. 51, nº 1150 |
| AZB1 | Aitzbitarte III | Western Pyrenees | Bison | msh | Own Review |
| AZB2 | Aitzbitarte III | Western Pyrenees | Aurochs | nch | Own Review |
| AZB3 | Aitzbitarte V | Western Pyrenees | Bison | str | Own Review |
| AZB4 | Aitzbitarte V | Western Pyrenees | Bison | str | Own Review |
| AZB5 | Aitzbitarte IX | Western Pyrenees | Bison | msh | Garate, 2018: fig. 6 |
| ALK1 | Alkerdi 2 | Western Pyrenees | Bison | str | Garate et al. 2017: p. 11, fig. 2 |
| BR1 | Brassempouy | Western Pyrenees | Bison | cnh | Fourloubey, 2019: p. 256, nºLT11 |
| CO13 | Cosquer | Mediterranean | Aurochs | cnh | Clottes et al. 2005: p. 129, Mé1 sect. 203 |
| CO66 | Cosquer | Mediterranean | Bison | str | Clottes et al. 2005: p. 115, fig. 96 |
| CO67 | Cosquer | Mediterranean | Aurochs | str | Clottes et al. 2005: p. 43, fig. 25 |
| CO68 | Cosquer | Mediterranean | Aurochs | str | Clottes et al. 2005: p. 134, fig. 121 |
| CO69 | Cosquer | Mediterranean | Aurochs | msh | Clottes et al. 2005: p. 91, fig. 70 |
| CO70 | Cosquer | Mediterranean | Aurochs | cnh | Clottes et al. 2005: p. 61, fig. 41 |
| CO71 | Cosquer | Mediterranean | Aurochs | msh | Clottes et al. 2005: p. 37, fig. 19 |
| CO72 | Cosquer | Mediterranean | Bison | str | Clottes et al. 2005 |
| CO73 | Cosquer | Mediterranean | Bison | cnh | Clottes et al. 2005: p. 88, fig. 66 |
| CO74 | Cosquer | Mediterranean | Bison | cnh | Clottes et al. 2005: p. 115, fig. 96 |
| CO75 | Cosquer | Mediterranean | Bison | cnh | Clottes et al. 2005: p. 113, fig. 94 |
| CU1 | Cussac | Perigord | Bison | msh | Aujoulat et al., 2002 |
| CU3 | Cussac | Perigord | Bison | msh | Aujoulat et al., 2002 |
| CU5 | Cussac | Perigord | Aurochs | msh | Aujoulat et al., 2002 |
| CU9 | Cussac | Perigord | Bison | str | Aujoulat et al., 2002 |
| CU10 | Cussac | Perigord | Aurochs | msh | Aujoulat et al., 2002 |
| CU11 | Cussac | Perigord | Bison | str | Aujoulat et al., 2002 |
| CU12 | Cussac | Perigord | Bison | msh | Aujoulat et al., 2002 |
| CU15 | Cussac | Perigord | Bison | nch | Aujoulat et al., 2002 |
| CU16 | Cussac | Perigord | Bison | msh | Aujoulat et al., 2002 |
| CU17 | Cussac | Perigord | Bison | nch | Aujoulat et al., 2002 |
| CU18 | Cussac | Perigord | Bison | str | Aujoulat et al., 2002 |
| CU19 | Cussac | Perigord | Bison | cnh | Aujoulat et al., 2002 |
| CU20 | Cussac | Perigord | Bison | msh | Aujoulat et al., 2002 |
| CU21 | Cussac | Perigord | Bison | str | Aujoulat et al., 2002 |
| CU22 | Cussac | Perigord | Aurochs | msh | Aujoulat et al., 2002 |
| ER1 | Erberua | Western Pyrenees | Bison | msh | Larribau, 2013: p. 320, nº30ddm |
| ER2 | Erberua | Western Pyrenees | Bison | msh | Larribau, 2013: p. 321, nº31ddm |
| GA4 | Gargas | Central Pyrenees | Bison | nch | Barrière, 1976 |
| GA5 | Gargas | Central Pyrenees | Bison | str | Barrière, 1976 |
| GA6 | Gargas | Central Pyrenees | Aurochs | msh | Barrière, 1976 |
| GA8 | Gargas | Central Pyrenees | Aurochs | cnh | Barrière, 1976 |
| GA9 | Gargas | Central Pyrenees | Aurochs | str | Barrière, 1976 |
| GA10 | Gargas | Central Pyrenees | Bison | msh | Barrière, 1976 |
| GA16 | Gargas | Central Pyrenees | Aurochs | cnh | Barrière, 1976 |
| GA17 | Gargas | Central Pyrenees | Bison | str | Barrière, 1976 |
| GA19 | Gargas | Central Pyrenees | Bison | str | Barrière, 1976 |
| GA20 | Gargas | Central Pyrenees | Bison | str | Barrière, 1976 |
| GA21 | Gargas | Central Pyrenees | Bison | cnh | Barrière, 1976 |
| GA22 | Gargas | Central Pyrenees | Bison | str | Barrière, 1976 |
| **Code** | **Site** | **Region** | **Motif** | **Convention**  **Horn** | **References** |
| GA23 | Gargas | Central Pyrenees | Bison | nch | Barrière, 1976 |
| GA24 | Gargas | Central Pyrenees | Bison | nho | Barrière, 1976 |
| GA29 | Gargas | Central Pyrenees | Bison | nch | Barrière, 1976 |
| GA30 | Gargas | Central Pyrenees | Bison | nho | Barrière, 1976 |
| GA31 | Gargas | Central Pyrenees | Bison | str | Barrière, 1976 |
| GA38 | Gargas | Central Pyrenees | Bison | str | Barrière, 1976 |
| GA40 | Gargas | Central Pyrenees | Bison | str | Barrière, 1976 |
| GA42 | Gargas | Central Pyrenees | Bison | nho | Barrière, 1976 |
| GA43 | Gargas | Central Pyrenees | Bison | nch | Barrière, 1976 |
| GA45 | Gargas | Central Pyrenees | Aurochs | msh | Barrière, 1976 |
| GA46 | Gargas | Central Pyrenees | Bison | str | Barrière, 1976 |
| GA47 | Gargas | Central Pyrenees | Bison | str | Barrière, 1976 |
| GA48 | Gargas | Central Pyrenees | Aurochs | cnh | Barrière, 1976 |
| GA49 | Gargas | Central Pyrenees | Bison | msh | Barrière, 1976 |
| GA50 | Gargas | Central Pyrenees | Bison | msh | Barrière, 1976 |
| GA51 | Gargas | Central Pyrenees | Bison | msh | Barrière, 1976 |
| GA52 | Gargas | Central Pyrenees | Bison | str | Barrière, 1976 |
| GA56 | Gargas | Central Pyrenees | Bison | cnh | Barrière, 1976 |
| GA58 | Gargas | Central Pyrenees | Bison | str | Barrière, 1976 |
| GA60 | Gargas | Central Pyrenees | Bison | msh | Barrière, 1976 |
| I1 | Isturitz | Western Pyrenees | Bison | cnh | Rivero and Garate, 2014: p.254, fig. 6 |
| I3 | Isturitz | Western Pyrenees | Bison | nho | Rivero and Garate, 2014: p.254, fig. 6 |
| I4 | Isturitz | Western Pyrenees | Bison | str | Rivero and Garate, 2014: p. 255, fig. 6 |
| I5 | Isturitz | Western Pyrenees | Aurochs | cnh | Rivero and Garate, 2014: p. 255, fig. 6 |
| I6 | Isturitz | Western Pyrenees | Bison | nho | Rivero and Garate, 2014: p. 255, fig. 6 |
| I17 | Isturitz | Western Pyrenees | Bison | cnh | Rivero and Garate, 2014: p. 257, fig. 9 |
| I18 | Isturitz | Western Pyrenees | Bison | str | Rivero and Garate, 2014: p. 257, fig. 9 |
| I19 | Isturitz | Western Pyrenees | Bison | msh | Rivero and Garate, 2014: p. 257, fig. 9 |
| I20 | Isturitz | Western Pyrenees | Bison | cnh | Rivero and Garate, 2014: p. 256, fig. 8 |
| I21 | Isturitz | Western Pyrenees | Bison | nho | Rivero and Garate, 2014: p. 262, fig. 18 |
| I22 | Isturitz | Western Pyrenees | Aurochs | cnh | Rivero and Garate, 2014: p. 262, fig. 19 |
| PA12 | Parpalló | Mediterranean | Aurochs | nho | Villaverde, 1994: vol. 2, p. 16127, nº16120A, fig. 21 |
| PM1 | Pech Merle | Quercy | Bison | nch | Lorblanchet, 2010: p. 68, fig. 12, B2 |
| PM3 | Pech Merle | Quercy | Aurochs | cnh | Lorblanchet, 2010: p. 68, fig. 12, A1 |
| PM4 | Pech Merle | Quercy | Aurochs | nho | Lorblanchet, 2010: p. 68, fig. 12, A3 |
| PM5 | Pech Merle | Quercy | Aurochs | cnh | Lorblanchet, 2010: p. 150, fig. 4 |
| PM6 | Pech Merle | Quercy | Aurochs | nch | Lorblanchet, 2010: p.202, fig. 20. I |
| PM9 | Pech Merle | Quercy | Bison | str | Lorblanchet, 2010: p.137, fig.1 |
| PER2 | Pergouset | Quercy | Bison | str | Lorblanchet, 2001: p. 109, fig.90, nº101 |
| PER3 | Pergouset | Quercy | Bison | nch | Lorblanchet, 2001: p.126, fig. 104 and 105 |
| RO2 | Rocadour | Quercy | Aurochs | nch | Lorblanchet et al., 2009: p. 36, Panneau IV, nº38 |
| RO5 | Rocadour | Quercy | Bison | cnh | Lorblanchet et al., 2009: p.49, Panneau VI, nº7 |
| RO6 | Rocadour | Quercy | Bison | cnh | Lorblanchet et al., 2009: p.49, Panneau VI, nº20 |
| TRF3 | Trois Frères | Central Pyrenees | Bison | cnh | Bégouën and Breuil, 1958: p. 26, fig. 27 |
| TRF4 | Trois Frères | Central Pyrenees | Bison | cnh | Bégouën and Breuil, 1958: p. 26, fig. 28 |
| TRF5 | Trois Frères | Central Pyrenees | Bison | msh | Bégouën and Breuil, 1958: p. 26, fig. 28 |
| TRF6 | Trois Frères | Central Pyrenees | Bison | cnh | Bégouën and Breuil, 1958: p. 28, fig.30 |
| **Code** | **Site** | **Region** | **Motif** | **Convention**  **Horn** | **References** |
| TRF7 | Trois Frères | Central Pyrenees | Bison | msh | Bégouën and Breuil, 1958: p. 26, fig. 28 |
| TRF8 | Trois Frères | Central Pyrenees | Aurochs | msh | Bégouën and Breuil, 1958: p. 26, fig. 28 |
| TRF9 | Trois Frères | Central Pyrenees | Aurochs | cnh | Bégouën and Breuil, 1958: p. 19, fig. 18 |

**S1 Table. List of represented bovid animals considered in the study.**
