# Supplementary material for: Redefining shared symbolic networks during the Gravettian in Western Europe: New data from the rock art findings in Aitzbitarte caves (Northern Spain)
Source: PLoS One. 2020 Oct 28;15(10):e0240481. doi: 10.1371/journal.pone.0240481 (PMC7592797; doi:10.1371/journal.pone.0240481)
Supplement: S2 Table — (DOCX) [file pone.0240481.s002.docx]

| **Code** | **Site** | **Region** | **Motif** | **Front legs** | **Back legs** | **References** |
| --- | --- | --- | --- | --- | --- | --- |
| ALB1 | Abri Labattut | Perigord | Horse | otf | otb | Airvaux et al., 1983: p. 240, fig. 5.3 |
| ALB2 | Abri Labattut | Perigord | Horse | ncf | rob | Airvaux et al., 1983: p. 240, fig. 5.3 |
| ALR1 | Abri Laraux | Perigord | Horse | ref | nbl | Airvaux et al., 1983: p. 236, fig. 1.1 |
| AZB1 | Aitzbitarte III | Western Pyrenees | Bison | ncf | msb | Own Review |
| AZB2 | Aitzbitarte III | Western Pyrenees | Aurochs | ref | nbl | Own Review |
| AZB3 | Aitzbitarte V | Western Pyrenees | Bison | nfl | nbl | Own Review |
| AZB4 | Aitzbitarte V | Western Pyrenees | Bison | nfl | nbl | Own Review |
| ALK1 | Alkerdi 2 | Western Pyrenees | Bison | pof | msb | Garate el al. 2017: p. 11, fig. 2 |
| BR2 | Brassempouy | Western Pyrenees | Bison | ref | nbl | Fourloubey, 2019: p. 256, nºLT11 |
| BR4 | Brassempouy | Western Pyrenees | Unindefined | nfl | reb | Fourloubey, 2019: p. 256, nºLT11 |
| CO1 | Cosquer | Mediterranean | Bird (Uncommon) | nfl | msb | Clottes et al. 2005: p. 44, fig. 26. Pi2 |
| CO2 | Cosquer | Mediterranean | Bird (Uncommon) | nfl | pob | Clottes et al. 2005: p. 44, fig. 26. Pi1 |
| CO3 | Cosquer | Mediterranean | Cervide  (Deer) | pof | pob | Clottes et al. 2005: p. 129, C1 sect. 123 |
| CO4 | Cosquer | Mediterranean | Cervide  (Hind) | pof | nbl | Clottes et al. 2005: p. 129, C2 sect. 108 |
| CO5 | Cosquer | Mediterranean | Cervide  (Hind) | pof | pob | Clottes et al. 2005: p. 129. C3 sect. 205 |
| CO6 | Cosquer | Mediterranean | Cervide  (Hind) | ncf | rob | Clottes et al. 2005: p. 129, C4 sect. 205 |
| CO7 | Cosquer | Mediterranean | Horse | nfl | nbl | Clottes et al. 2005: p. 129, Chv28 |
| CO8 | Cosquer | Mediterranean | Cervide  (Deer) | ncf | ncb | Clottes et al. 2005: p. 129, C5 sect 201 |
| CO9 | Cosquer | Mediterranean | Cervide  (Deer) | msf | ncb | Clottes et al. 2005: p. 129, C6 sect. 107 |
| CO10 | Cosquer | Mediterranean | Cervide  (Deer) | nfl | nbl | Clottes et al. 2005: p. 129, C7 sect. 104 |
| CO11 | Cosquer | Mediterranean | Cervide  (Deer) | nfl | nbl | Clottes et al. 2005: p. 129, C10 sect. 101 |
| CO12 | Cosquer | Mediterranean | Cervide  (Hind) | msf | nbl | Clottes et al. 2005: p. 129, C12 sect. 106 |
| CO13 | Cosquer | Mediterranean | Aurochs | msf | msb | Clottes et al. 2005: p. 129, Mé1 sect. 203 |
| CO14 | Cosquer | Mediterranean | Cervide  (Deer) | pof | ncb | Clottes et al. 2005: p. 129, Mé2 sect. 108 |
| CO15 | Cosquer | Mediterranean | Horse | ncf | ncb | Clottes et al. 2005: p. 123, Bq6 sect. 108 |
| CO16 | Cosquer | Mediterranean | Horse | ncf | ncb | Clottes et al. 2005: p. 123, Bq24 sect. 108 |
| CO17 | Cosquer | Mediterranean | Ibex | rof | rob | Clottes et al. 2005: p. 123, Bq1 sect. 123 |
| CO18 | Cosquer | Mediterranean | Ibex | pof | ncb | Clottes et al. 2005: p. 123, Bq2 sect. 108 |
| CO19 | Cosquer | Mediterranean | Ibex | ncf | pob | Clottes et al. 2005: p. 123, Bq3 sect. 108 |
| CO20 | Cosquer | Mediterranean | Ibex | pof | ncb | Clottes et al. 2005: p. 123, Bq4 sect. 205 |
| CO21 | Cosquer | Mediterranean | Ibex | ncf | pob | Clottes et al. 2005: p. 123, Bq5 sect 205 |
| CO22 | Cosquer | Mediterranean | Unidentified | ncf | msb | Clottes et al. 2005: p. 123, Bq6 sect. 108 |
| CO23 | Cosquer | Mediterranean | Ibex | pof | ncb | Clottes et al. 2005: p. 123, Bq7 sect. 204 |
| CO24 | Cosquer | Mediterranean | Ibex | ncf | ncb | Clottes et al. 2005: p. 123, Bq8 sect. 202 |
| CO25 | Cosquer | Mediterranean | Ibex | ncf | nbl | Clottes et al. 2005: p. 123, Bq9 sect. 107 |
| CO26 | Cosquer | Mediterranean | Ibex | pof | nbl | Clottes et al. 2005: p. 123, Bq10 sect. 101 |
| CO27 | Cosquer | Mediterranean | Ibex | nfl | nbl | Clottes et al. 2005: p. 123, Bq11 sect. 101 |
| CO28 | Cosquer | Mediterranean | Ibex | ncf | ncb | Clottes et al. 2005: p. 123, Bq12 sect. 108 |
| CO29 | Cosquer | Mediterranean | Ibex | ncf | nbl | Clottes et al. 2005: p. 123, Bq14 sect. 102 |
| **Code** | **Site** | **Region** | **Motif** | **Front legs** | **Back legs** | **References** |
| CO30 | Cosquer | Mediterranean | Ibex | pof | pob | Clottes et al. 2005: p. 123, Bq15 sect. 205 |
| CO31 | Cosquer | Mediterranean | Ibex | pof | ncb | Clottes et al. 2005: p. 123, Bq16 sect. 107 |
| CO32 | Cosquer | Mediterranean | Ibex | ncf | pob | Clottes et al. 2005: p. 123, Bq17 sect. 101 |
| CO33 | Cosquer | Mediterranean | Ibex | ncf | ncb | Clottes et al. 2005: p. 123, Bq19 sect. 107 |
| CO34 | Cosquer | Mediterranean | Ibex | nfl | nbl | Clottes et al. 2005: p. 123, Bq20 sect. 107 |
| CO35 | Cosquer | Mediterranean | Ibex | nfl | nbl | Clottes et al. 2005: p. 123, Bq20b sect. 101 |
| CO36 | Cosquer | Mediterranean | Ibex | nfl | nbl | Clottes et al. 2005: p. 123, Bq22 sect. 204 |
| CO37 | Cosquer | Mediterranean | Ibex | ncf | pob | Clottes et al. 2005: p. 123, Chm1 sect. 108 |
| CO38 | Cosquer | Mediterranean | Ibex | msf | ncb | Clottes et al. 2005: p. 123, Chm2 sect. 205 |
| CO39 | Cosquer | Mediterranean | Ibex | ncf | pob | Clottes et al. 2005: p. 123, Chm4 sect. 102 |
| CO40 | Cosquer | Mediterranean | Ibex | msf | msb | Clottes et al. 2005: p. 123, Chm5 sect. 102 |
| CO41 | Cosquer | Mediterranean | Horse | ncf | ncb | Clottes et al. 2005: p. 110, Chv3 sect. 101 |
| CO42 | Cosquer | Mediterranean | Horse | nfl | nbl | Clottes et al. 2005: p. 110, Chv4 sect. 101 |
| CO43 | Cosquer | Mediterranean | Horse | ref | nbl | Clottes et al. 2005: p. 110, Chv5 sect. 101 |
| CO44 | Cosquer | Mediterranean | Horse | nfl | nbl | Clottes et al. 2005: p. 110, Chv6 sect. 101 |
| CO45 | Cosquer | Mediterranean | Horse | msf | pob | Clottes et al. 2005: p. 110, Chv7 sect. 123 |
| CO46 | Cosquer | Mediterranean | Horse | ncf | ncb | Clottes et al. 2005: p. 110, Chv8 sect. 123 |
| CO47 | Cosquer | Mediterranean | Horse | nfl | nbl | Clottes et al. 2005: p. 110, Chv9 sect. 117 |
| CO48 | Cosquer | Mediterranean | Horse | nfl | nbl | Clottes et al. 2005: p. 110, Chv10 sect. 108 |
| CO49 | Cosquer | Mediterranean | Horse | msf | msb | Clottes et al. 2005: p. 110, Chv11 sect. 108 |
| CO50 | Cosquer | Mediterranean | Horse | ncf | ncb | Clottes et al. 2005: p. 110, Chv13 sect. 204 |
| CO51 | Cosquer | Mediterranean | Horse | ncf | nbl | Clottes et al. 2005: p. 110, Chv14 sect. 203 |
| CO52 | Cosquer | Mediterranean | Horse | ncf | nbl | Clottes et al. 2005: p. 110, Chv15 sect. 204 |
| CO53 | Cosquer | Mediterranean | Horse | msf | nbl | Clottes et al. 2005: p. 110, Chv12 sect. 204 |
| CO54 | Cosquer | Mediterranean | Horse | nfl | nbl | Clottes et al. 2005: p. 110, Chv18 sect. 101 |
| CO55 | Cosquer | Mediterranean | Horse | msf | nbl | Clottes et al. 2005: p. 110, Chv19 sect. 108 |
| CO56 | Cosquer | Mediterranean | Horse | msf | nbl | Clottes et al. 2005: p. 110, Chv20 sect. 202 |
| CO57 | Cosquer | Mediterranean | Horse | msf | pob | Clottes et al. 2005: p. 110, Chv24 sect. 205 |
| CO58 | Cosquer | Mediterranean | Horse | msf | msb | Clottes et al. 2005: p. 110, Chv25 sect. 107 |
| CO59 | Cosquer | Mediterranean | Horse | ncf | nbl | Clottes et al. 2005: p. 111, Chv31 sect. 205 |
| CO60 | Cosquer | Mediterranean | Horse | nfl | nbl | Clottes et al. 2005: p. 111, Chv32 sect. 107 |
| CO61 | Cosquer | Mediterranean | Horse | nfl | nbl | Clottes et al. 2005: p. 111, Chv34 sect. 102 |
| CO62 | Cosquer | Mediterranean | Horse | ncf | ncb | Clottes et al. 2005: p. 111, Chv38 sect. 102 |
| CO63 | Cosquer | Mediterranean | Horse | rof | nbl | Clottes et al. 2005: p. 111, Chv39 sect. 102 |
| CO64 | Cosquer | Mediterranean | Horse | msf | nbl | Clottes et al. 2005: p. 111, Chv42 sect. 204 |
| CO65 | Cosquer | Mediterranean | Horse | rof | ncb | Clottes et al. 2005: p. 111, Chv61 sect. 203 |
| CO66 | Cosquer | Mediterranean | Bison | ncf | ncb | Clottes et al. 2005: p. 115, fig. 96 |
| CO67 | Cosquer | Mediterranean | Aurochs | nfl | nbl | Clottes et al. 2005: p. 43, fig. 25 |
| CO68 | Cosquer | Mediterranean | Aurochs | pof | nbl | Clottes et al. 2005: p. 134, fig. 121 |
| **Code** | **Site** | **Region** | **Motif** | **Front legs** | **Back legs** | **References** |
| CO69 | Cosquer | Mediterranean | Aurochs | nfl | nbl | Clottes et al. 2005: p. 91, fig. 70 |
| CO70 | Cosquer | Mediterranean | Aurochs | ncf | nbl | Clottes et al. 2005: p. 61, fig. 41 |
| CO71 | Cosquer | Mediterranean | Aurochs | nfl | nbl | Clottes et al. 2005: p. 37, fig. 19 |
| CO72 | Cosquer | Mediterranean | Bison | ncf | nbl | Clottes et al. 2005 |
| CO73 | Cosquer | Mediterranean | Bison | nfl | nbl | Clottes et al. 2005: p. 88, fig. 66 |
| CO74 | Cosquer | Mediterranean | Bison | nfl | nbl | Clottes et al. 2005: p. 115, fig. 96 |
| CO75 | Cosquer | Mediterranean | Bison | ncf | nbl | Clottes et al. 2005: p. 113, fig. 94 |
| CU1 | Cussac | Perigord | Bison | ref | nbl | Aujoulat et al., 2002 |
| CU2 | Cussac | Perigord | Horse | rof | rob | Aujoulat et al., 2002 |
| CU3 | Cussac | Perigord | Bison | nfl | nbl | Aujoulat et al., 2002 |
| CU4 | Cussac | Perigord | Mammoth (Uncommon) | pof | reb | Aujoulat et al., 2002 |
| CU5 | Cussac | Perigord | Aurochs | msf | ncb | Aujoulat et al., 2002 |
| CU6 | Cussac | Perigord | Mammoth (Uncommon) | pof | ncb | Aujoulat et al., 2002 |
| CU7 | Cussac | Perigord | Mammoth (Uncommon) | msf | pob | Aujoulat et al., 2002 |
| CU8 | Cussac | Perigord | Horse | rof | ncb | Aujoulat et al., 2002 |
| CU9 | Cussac | Perigord | Bison | ref | rob | Aujoulat et al., 2002 |
| CU10 | Cussac | Perigord | Aurochs | ncf | reb | Aujoulat et al., 2002 |
| CU11 | Cussac | Perigord | Bison | rof | pob | Aujoulat et al., 2002 |
| CU12 | Cussac | Perigord | Bison | rof | nbl | Aujoulat et al., 2002 |
| CU13 | Cussac | Perigord | Horse | rof | nbl | Aujoulat et al., 2002 |
| CU14 | Cussac | Perigord | Horse | pof | rob | Aujoulat et al., 2002 |
| CU15 | Cussac | Perigord | Bison | rof | reb | Aujoulat et al., 2002 |
| CU16 | Cussac | Perigord | Bison | nfl | reb | Aujoulat et al., 2002 |
| CU17 | Cussac | Perigord | Bison | nfl | reb | Aujoulat et al., 2002 |
| CU18 | Cussac | Perigord | Bison | nfl | nbl | Aujoulat et al., 2002 |
| CU19 | Cussac | Perigord | Bison | nfl | nbl | Aujoulat et al., 2002 |
| CU20 | Cussac | Perigord | Bison | nfl | nbl | Aujoulat et al., 2002 |
| CU21 | Cussac | Perigord | Bison | nfl | nbl | Aujoulat et al., 2002 |
| CU22 | Cussac | Perigord | Aurochs | ncf | nbl | Aujoulat et al., 2002 |
| EP1 | El Pendo | Western Pyrenees | Unidentified | ref | nbl | Rivero et al., submitted |
| GA1 | Gargas | Central Pyrenees | Horse | rof | rob | Barrière, 1976 |
| GA2 | Gargas | Central Pyrenees | Horse | nfl | nbl | Barrière, 1976 |
| GA3 | Gargas | Central Pyrenees | Horse | rof | rob | Barrière, 1976 |
| GA4 | Gargas | Central Pyrenees | Bison | ncf | nbl | Barrière, 1976 |
| GA5 | Gargas | Central Pyrenees | Bison | nfl | nbl | Barrière, 1976 |
| GA6 | Gargas | Central Pyrenees | Aurochs | nfl | nbl | Barrière, 1976 |
| GA7 | Gargas | Central Pyrenees | Unidentified | nfl | rob | Barrière, 1976 |
| GA8 | Gargas | Central Pyrenees | Aurochs | nfl | nbl | Barrière, 1976 |
| GA9 | Gargas | Central Pyrenees | Aurochs | nfl | nbl | Barrière, 1976 |
| **Code** | **Site** | **Region** | **Motif** | **Front legs** | **Back legs** | **References** |
| GA10 | Gargas | Central Pyrenees | Bison | nfl | nbl | Barrière, 1976 |
| GA11 | Gargas | Central Pyrenees | Ibex | rof | rob | Barrière, 1976 |
| GA12 | Gargas | Central Pyrenees | Cervide | ref | reb | Barrière, 1976 |
| GA13 | Gargas | Central Pyrenees | Unidentified | ref | otb | Barrière, 1976 |
| GA14 | Gargas | Central Pyrenees | Ibex | nfl | nbl | Barrière, 1976 |
| GA15 | Gargas | Central Pyrenees | Ibex | nfl | nbl | Barrière, 1976 |
| GA16 | Gargas | Central Pyrenees | Aurochs | msf | ncb | Barrière, 1976 |
| GA17 | Gargas | Central Pyrenees | Bison | nfl | nbl | Barrière, 1976 |
| GA18 | Gargas | Central Pyrenees | Mammoth (Uncommon) | ncf | ncb | Barrière, 1976 |
| GA19 | Gargas | Central Pyrenees | Bison | nfl | nbl | Barrière, 1976 |
| GA20 | Gargas | Central Pyrenees | Bison | nfl | nbl | Barrière, 1976 |
| GA21 | Gargas | Central Pyrenees | Bison | nfl | nbl | Barrière, 1976 |
| GA22 | Gargas | Central Pyrenees | Bison | ncf | nbl | Barrière, 1976 |
| GA23 | Gargas | Central Pyrenees | Bison | nfl | nbl | Barrière, 1976 |
| GA24 | Gargas | Central Pyrenees | Bison | nfl | nbl | Barrière, 1976 |
| GA25 | Gargas | Central Pyrenees | Horse | nfl | nbl | Barrière, 1976 |
| GA26 | Gargas | Central Pyrenees | Horse | nfl | nbl | Barrière, 1976 |
| GA27 | Gargas | Central Pyrenees | Horse | nfl | nbl | Barrière, 1976 |
| GA28 | Gargas | Central Pyrenees | Horse | ncf | nbl | Barrière, 1976 |
| GA29 | Gargas | Central Pyrenees | Bison | rof | otb | Barrière, 1976 |
| GA30 | Gargas | Central Pyrenees | Bison | rof | ncb | Barrière, 1976 |
| GA31 | Gargas | Central Pyrenees | Bison | nfl | nbl | Barrière, 1976 |
| GA32 | Gargas | Central Pyrenees | Horse | ref | nbl | Barrière, 1976 |
| GA33 | Gargas | Central Pyrenees | Horse | pof | nbl | Barrière, 1976 |
| GA34 | Gargas | Central Pyrenees | Horse | pof | msb | Barrière, 1976 |
| GA35 | Gargas | Central Pyrenees | Horse | nfl | nbl | Barrière, 1976 |
| GA36 | Gargas | Central Pyrenees | Ibex | ncf | nbl | Barrière, 1976 |
| GA37 | Gargas | Central Pyrenees | Cervide | ref | msb | Barrière, 1976 |
| GA38 | Gargas | Central Pyrenees | Bison | nfl | nbl | Barrière, 1976 |
| GA39 | Gargas | Central Pyrenees | Horse | ref | nbl | Barrière, 1976 |
| GA40 | Gargas | Central Pyrenees | Bison | msf | ncb | Barrière, 1976 |
| GA41 | Gargas | Central Pyrenees | Horse | msf | ncb | Barrière, 1976 |
| GA42 | Gargas | Central Pyrenees | Bison | ref | pob | Barrière, 1976 |
| GA43 | Gargas | Central Pyrenees | Bison | rof | nbl | Barrière, 1976 |
| GA44 | Gargas | Central Pyrenees | Horse | pof | nbl | Barrière, 1976 |
| GA45 | Gargas | Central Pyrenees | Aurochs | ref | nbl | Barrière, 1976 |
| GA46 | Gargas | Central Pyrenees | Bison | nfl | nbl | Barrière, 1976 |
| GA47 | Gargas | Central Pyrenees | Bison | nfl | nbl | Barrière, 1976 |
| GA48 | Gargas | Central Pyrenees | Aurochs | nfl | nbl | Barrière, 1976 |
| **Code** | **Site** | **Region** | **Motif** | **Front legs** | **Back legs** | **References** |
| GA49 | Gargas | Central Pyrenees | Bison | ncf | ncb | Barrière, 1976 |
| GA50 | Gargas | Central Pyrenees | Bison | nfl | nbl | Barrière, 1976 |
| GA51 | Gargas | Central Pyrenees | Bison | nfl | nbl | Barrière, 1976 |
| GA52 | Gargas | Central Pyrenees | Bison | nfl | nbl | Barrière, 1976 |
| GA53 | Gargas | Central Pyrenees | Ibex | pof | nbl | Barrière, 1976 |
| GA54 | Gargas | Central Pyrenees | Unidentified | nfl | nbl | Barrière, 1976 |
| GA55 | Gargas | Central Pyrenees | Unidentified | nfl | nbl | Barrière, 1976 |
| GA56 | Gargas | Central Pyrenees | Bison | nfl | nbl | Barrière, 1976 |
| GA57 | Gargas | Central Pyrenees | Mammoth (Uncommon) | otf | ncb | Barrière, 1976 |
| GA58 | Gargas | Central Pyrenees | Bison | pof | nbl | Barrière, 1976 |
| GA59 | Gargas | Central Pyrenees | Feline  (Uncommon) | rof | ncb | Barrière, 1976 |
| GA60 | Gargas | Central Pyrenees | Bison | msf | ncb | Barrière, 1976 |
| I1 | Isturitz | Western Pyrenees | Bison | msf | nbl | Rivero and Garate, 2014: p.254, fig. 6 |
| I2 | Isturitz | Western Pyrenees | Horse | nfl | nbl | Rivero and Garate, 2014: p.254, fig. 6 |
| I3 | Isturitz | Western Pyrenees | Bison | ncf | reb | Rivero and Garate, 2014: p. 255, fig. 6 |
| I4 | Isturitz | Western Pyrenees | Bison | otf | nbl | Rivero and Garate, 2014: p. 255, fig. 6 |
| I5 | Isturitz | Western Pyrenees | Unidentified | ncf | reb | Rivero and Garate, 2014: p. 255, fig. 6 |
| I6 | Isturitz | Western Pyrenees | Bison | ref | nbl | Rivero and Garate, 2014: p. 255, fig. 6 |
| I7 | Isturitz | Western Pyrenees | Unidentified | msf | pob | Rivero and Garate, 2014: p. 255, fig. 6 |
| I8 | Isturitz | Western Pyrenees | Unidentified | ref | nbl | Own review |
| I9 | Isturitz | Western Pyrenees | Unidentified | rof | nbl | Own review |
| I10 | Isturitz | Western Pyrenees | Unidentified | nfl | reb | Own review |
| I11 | Isturitz | Western Pyrenees | Unidentified | rof | nbl | Own review |
| I12 | Isturitz | Western Pyrenees | Aurochs | ncf | rob | Rivero and Garate, 2014: p. 255, fig. 6 |
| I13 | Isturitz | Western Pyrenees | Mammoth (Uncommon) | rof | reb | Rivero and Garate, 2014: p. 255, fig. 6 |
| I14 | Isturitz | Western Pyrenees | Mammoth (Uncommon) | ncf | nbl | Rivero and Garate, 2014: p. 255, fig. 6 |
| I15 | Isturitz | Western Pyrenees | Cervide  (Deer) | rof | reb | Rivero and Garate, 2014: p. 265, fig. 24 |
| I16 | Isturitz | Western Pyrenees | Unidentified | rof | rob | Rivero and Garate, 2014: p. 265, fig. 24 |
| I17 | Isturitz | Western Pyrenees | Bison | ref | reb | Rivero and Garate, 2014: p. 257, fig. 9 |
| I18 | Isturitz | Western Pyrenees | Bison | otf | reb | Rivero and Garate, 2014: p. 257, fig. 9 |
| I19 | Isturitz | Western Pyrenees | Bison | nfl | reb | Rivero and Garate, 2014: p. 257, fig. 9 |
| I20 | Isturitz | Western Pyrenees | Bison | ref | ncb | Rivero and Garate, 2014: p. 256, fig. 8 |
| I21 | Isturitz | Western Pyrenees | Bison | msf | rob | Rivero and Garate, 2014: p. 262, fig. 18 |
| I22 | Isturitz | Western Pyrenees | Aurochs | msf | ncb | Rivero and Garate, 2014: p. 262, fig. 19 |
| I23 | Isturitz | Western Pyrenees | Cervide  (Deer) | ref | nbl | Rivero and Garate, 2014: p. 264, fig. .22 |
| LP1 | Le Portel | Central Pyrenees | Horse | pof | pob | Beltrán et al., 1966: p. 86, fig. XXXIX |
| LP2 | Le Portel | Central Pyrenees | Horse | nfl | pob | Beltrán et al., 1966: p. 107, fig. LI |
| LP3 | Le Portel | Central Pyrenees | Horse | nfl | pob | Beltrán et al., 1966: p. 116, fig. LVII |
| PA1 | Parpalló | Mediterranean | Cervide  (Biche) | ncf | pob | Villaverde, 1994: vol. 2, p. 16191, nº16180A, fig. 37 |
| **Code** | **Site** | **Region** | **Motif** | **Front legs** | **Back legs** | **References** |
| PA2 | Parpalló | Mediterranean | Cervide  (Biche) | ncf | pob | Villaverde, 1994: vol. 2, p. 16191, nº16180A, fig. 37 |
| PA3 | Parpalló | Mediterranean | Ibex | ncf | pob | Villaverde, 1994: vol. 2, p. 16191, n 16169, fig. 30 |
| PA4 | Parpalló | Mediterranean | Ibex | ncf | pob | Villaverde, 1994: vol. 2, p. 16324, nº16342, fig. 67 |
| PA5 | Parpalló | Mediterranean | Horse | nfl | pob | Villaverde, 1994: vol. 2, p. 16127, nº16122A, fig. 23 |
| PA6 | Parpalló | Mediterranean | Horse | otf | msb | Villaverde, 1994: vol. 2, p.16127, nº16113A, fig. 36 |
| PA7 | Parpalló | Mediterranean | Ibex | rof | rob | Villaverde, 1994: vol. 2, p.16078, nº16061A, fig. 10 |
| PA8 | Parpalló | Mediterranean | Horse | msf | pob | Villaverde, 1994: vol. 2, p. 17724, nº17757B, fig. 140 |
| PA9 | Parpalló | Mediterranean | Horse | pof | pob | Villaverde, 1994: vol. 2, p. 17724, nº17758, fig. 141 |
| PA10 | Parpalló | Mediterranean | Unidentified | nfl | pob | Villaverde, 1994: vol. 2, p. 18185, nº18169, fig. 160 |
| PA11 | Parpalló | Mediterranean | Ibex | ref | pob | Villaverde, 1994: vol. 2, p. 16599, nº16569, fig. 82 |
| PA12 | Parpalló | Mediterranean | Aurochs | msf | nbl | Villaverde, 1994: vol. 2, p. 16127, nº16120A, fig. 21 |
| PA13 | Parpalló | Mediterranean | Cervide  (Deer) | ncf | ncb | Villaverde, 1994: vol. 2, p. 16021, nº16004, fig. 1 |
| PA14 | Parpalló | Mediterranean | Unidentified | rof | ncb | Villaverde, 1994: vol. 2, p. 16022, nº16036A, fig. 6 |
| PA15 | Parpalló | Mediterranean | Ibex | ncf | reb | Villaverde, 1994: vol. 2, p. 16022, nº16036B, fig. 7 |
| PA16 | Parpalló | Mediterranean | Unidentified | rof | nbl | Villaverde, 1994: vol. 2, p. 16078, nº16064, fig. 11 |
| PA17 | Parpalló | Mediterranean | Unidentified | rof | rbl | Villaverde, 1994: vol. 2, p. 16078, nº16069A, fig. 11 |
| PA18 | Parpalló | Mediterranean | Unidentified | nfl | rbl | Villaverde, 1994: vol. 2, p. 16078, nº16069B, fig. 11 |
| PA19 | Parpalló | Mediterranean | Unidentified | rfl | nbl | Villaverde, 1994: vol. 2, p. 16247, nº16224, fig. 43 |
| PA20 | Parpalló | Mediterranean | Ibex | rfl | nbl | Villaverde, 1994: vol. 2, p. 16323, nº16320, fig. 61 |
| PA21 | Parpalló | Mediterranean | Horse | rfl | nbl | Villaverde, 1994: vol. 2, p. 16324, nº16330A, fig. 62 |
| PA22 | Parpalló | Mediterranean | Ibex | rfl | rbl | Villaverde, 1994: vol. 2, p. 16324, nº16330B, fig. 63 |
| PM1 | Pech Merle | Quercy | Bison | rof | pob | Lorblanchet, 2010: p. 68, fig. 12, B2 |
| PM2 | Pech Merle | Quercy | Mammoth (Uncommon) | nfl | pob | Lorblanchet, 2010: p.62, fig. 8 |
| PM3 | Pech Merle | Quercy | Aurochs | ncf | rob | Lorblanchet, 2010: p. 68, fig. 12, A1 |
| PM4 | Pech Merle | Quercy | Aurochs | pof | pob | Lorblanchet, 2010: p. 68, fig. 12, A3 |
| PM5 | Pech Merle | Quercy | Aurochs | rof | ncb | Lorblanchet, 2010: p. 150, fig. 4 |
| PM6 | Pech Merle | Quercy | Ibex | msf | ncb | Lorblanchet, 2010: p.202, fig. 20. I |
| PM7 | Pech Merle | Quercy | Aurochs | nfl | pob | Lorblanchet, 2010: p. 138, fig. 2 (photo) |
| PM8 | Pech Merle | Quercy | Ibex | msf | ncb | Lorblanchet, 2010: p.202, fig. 20. III |
| PM9 | Pech Merle | Quercy | Bison | ncf | ncb | Lorblanchet, 2010: p.137, fig.1 |
| PM10 | Pech Merle | Quercy | Horse | msf | ncb | Lorblanchet, 2010: p. 107, fig. 2 |
| PM11 | Pech Merle | Quercy | Horse | rof | ncb | Lorblanchet, 2010: p. 107, fig. 2 |
| PM12 | Pech Merle | Quercy | Horse | ncf | ncb | Lorblanchet, 2010: p. 68, fig. 12, B1 |
| PER1 | Pergouset | Quercy | Cervide  (Biche) | msf | ncb | Lorblanchet, 2001: p. 106, fig. 87, nº100 |
| PER2 | Pergouset | Quercy | Bison | nfl | nbl | Lorblanchet, 2001: p. 109, fig.90, nº101 |
| PER3 | Pergouset | Quercy | Bison | nfl | nbl | Lorblanchet, 2001: p.126, fig. 104 and 105 |
| RO1 | Roucadour | Quercy | Cervide  (Megacero) | msf | ncb | Lorblanchet et al., 2009: p. 36, Panneau IV, nº39 |
| RO2 | Roucadour | Quercy | Bison | pof | ncb | Lorblanchet et al., 2009: p. 36, Panneau IV, nº38 |
| RO3 | Roucadour | Quercy | Cervide  (Megacero) | ref | ncb | Lorblanchet et al., 2009: p. 36, Panneau IV, nº48 |
| **Code** | **Site** | **Region** | **Motif** | **Front legs** | **Back legs** | **References** |
| RO 4 | Roucadour | Quercy | Cervide  (Megacero) | pof | ncb | Lorblanchet et al., 2009: p.49, Panneau VI, nº18 |
| RO5 | Roucadour | Quercy | Bison | ref | ncb | Lorblanchet et al., 2009: p.49, Panneau VI, nº7 |
| RO6 | Roucadour | Quercy | Bison | rof | ncb | Lorblanchet et al., 2009: p.49, Panneau VI, nº20 |
| RO7 | Roucadour | Quercy | Cervide  (Megacero) | pof | ncb | Lorblanchet et al., 2009: p.50, Panneau VI, nº35 |
| RO8 | Roucadour | Quercy | Horse | pof | ncb | Lorblanchet, 2010: p. 344, fig. 10a |
| RO9 | Roucadour | Quercy | Horse | pof | ncb | Lorblanchet et al., 2009: p. 60, Panneau VII, nº2 |
| RO10 | Roucadour | Quercy | Cervide  (Megacero) | rof | ncb | Lorblanchet, 2010: p. 345, fig. 11a |
| RO11 | Roucadour | Quercy | Cervide  (Megacero) | otf | ncb | Lorblanchet, 2010: p. 345, fig. 11a |
| RO12 | Roucadour | Quercy | Cervide  (Megacero) | ncf | pob | Lorblanchet et al., 2009: p. 46, Panneau IV, nº41 |
| RO13 | Roucadour | Quercy | Cervide  (Megacero) | pof | ncb | Lorblanchet, 2010: p.345, fig. 11b |
| TRF1 | Trois Frères | Central Pyrenees | Unidentified | pof | nbl | Bégouën and Breuil, 1958: p. 20, fig. 19 |
| TRF2 | Trois Frères | Central Pyrenees | Unidentified | nfl | reb | Bégouën and Breuil, 1958: p. 22, fig. 20 |
| TRF3 | Trois Frères | Central Pyrenees | Bison | nfl | nbl | Bégouën and Breuil, 1958: p. 26, fig. 27 |
| TRF4 | Trois Frères | Central Pyrenees | Bison | nfl | nbl | Bégouën and Breuil, 1958: p. 26, fig. 28 |
| TRF5 | Trois Frères | Central Pyrenees | Bison | nfl | nbl | Bégouën and Breuil, 1958: p. 26, fig. 28 |
| TRF6 | Trois Frères | Central Pyrenees | Bison | nfl | nbl | Bégouën and Breuil, 1958: p. 28, fig.30 |
| TFR7 | Trois Frères | Central Pyrenees | Bison | nfl | nbl | Bégouën and Breuil, 1958: p. 26, fig. 28 |
| TRF8 | Trois Frères | Central Pyrenees | Aurochs | nfl | nbl | Bégouën and Breuil, 1958: p. 26, fig. 28 |
| TRF9 | Trois Frères | Central Pyrenees | Aurochs | nfl | nbl | Bégouën and Breuil, 1958: p. 19, fig. 18 |
|  |  |  |  |  |  |  |

**S2 Table. List of animals with the legs represented that have been considered in the study.**
