# Supplementary material for: Redefining shared symbolic networks during the Gravettian in Western Europe: New data from the rock art findings in Aitzbitarte caves (Northern Spain)
Source: PLoS One. 2020 Oct 28;15(10):e0240481. doi: 10.1371/journal.pone.0240481 (PMC7592797; doi:10.1371/journal.pone.0240481)
Supplement: S3 Table — (DOCX) [file pone.0240481.s003.docx]

| **Site** | **D/C** | **Lab code** | **Sample description** | **Age (yr BP)** | **Error** | **Age (Cal BP)** | | **Reference** |
| --- | --- | --- | --- | --- | --- | --- | --- | --- |
|  |  |  |  |  |  | Max | Min |  |
| Cosquer | D | GifA96074 | Oval shape SIG100 | 28,370 | 440 | 33,462 | 31,374 | Valladas *et al*., 2017 |
| Cosquer | D | *GifA14228/SacA39210* | *HAF (Acid humic fraction)* | *28,060* | *550* | *33,404* | *31,106* | Valladas *et al*., 2017 |
| Cosquer | D | GifA96073 | Hand stencil MNN019 | 27,740 | 410 | 32,766 | 31,014 | Valladas *et al*., 2017 |
| Cosquer | D | GifA95195 | Bison BIS002 | 27,350 | 430 | 32,425 | 30,730 | Valladas *et al*., 2017 |
| Cosquer | D | GifA92409 | Hand stencil MNR007 | 27,110 | 400 | 31,885 | 30,531 | Valladas *et al*., 2017 |
| Cosquer | D | GifA92491 | Hand stencil MNR007 | 27,110 | 350 | 31,650 | 30,651 | Valladas *et al*., 2017 |
| Cosquer | D | GifA14172/SacA39203 | Hand stencil MNN001 | 26,900 | 290 | 31,365 | 30,620 | Valladas *et al*., 2017 |
| Cosquer | D | GifA14173/SacA39204 | Hand stencil MNN009 | 26,310 | 270 | 31,029 | 29,868 | Valladas *et al*., 2017 |
| Cosquer | D | GifA96069 | Bison BIS002 | 26,250 | 350 | 31,053 | 29,659 | Valladas *et al*., 2017 |
| Cosquer | D | GifA14157/SacA39220 | Bison BIS002 | 26,240 | 270 | 30,988 | 29,796 | Valladas *et al*., 2017 |
| Cosquer | D | *GifA92424* | *HAF (Acid humic fraction)* | *26,180* | *330* | *30,997* | *29,633* | Valladas *et al*., 2017 |
| Cosquer | D | GifA14167/SacA39199 | Animal shape AIN015 | 25,650 | 250 | 31,607 | 30,990 | Valladas *et al*., 2017 |
| Cosquer | D | GifA13418/SacA37400 | Horse CHV001 | 25,450 | 190 | 30,198 | 29,040 | Valladas *et al*., 2017 |
| Cosquer | D | GifA14162/SacA39194 | Line in a pilar SIG125 | 25,260 | 960 | 31,228 | 27,713 | Valladas *et al*., 2017 |
| Cosquer | D | GifA95358 | Hand stencil MNN012 | 24,840 | 340 | 29,701 | 28,136 | Valladas *et al*., 2017 |
| Cosquer | D | GifA96072 | Horse CHV005 | 24,730 | 300 | 29,480 | 28,122 | Valladas *et al*., 2017 |
| Cosquer | D | *GifA13484/SacA27402* | *HAF (Acid humic fraction)* | *24,340* | *170* | *28,742* | *27,965* | Valladas *et al*., 2017 |
| Cosquer | D | GifA14163/SacA39195 | Phallic pillar SIG119 | 23,830 | 210 | 28,392 | 27,578 | Valladas *et al*., 2017 |
| Cosquer | D | *GifA95372* | *HAF (Acid humic fraction)* | *23,150* | *620* | *28,577* | *26,150* | Valladas *et al*., 2017 |
| Cosquer | D | GifA13480/SacA37399 | Horse CHV005 | 22,920 | 160 | 27,581 | 26,871 | Valladas *et al*., 2017 |
| Cosquer | D | *GifA13485/SacA37403* | *HAF (Acid humic fraction)* | *22,860* | *330* | *27,711* | *26,430* | Valladas *et al*., 2017 |
| Cosquer | D | GifA13479/SacA37398 | Horse CHV005 | 22,440 | 130 | 27,155 | 26,350 | Valladas *et al*., 2017 |
| Cosquer | D | GifA14160/SacA39192 | Bison BIS005 | 20,120 | 510 | 25,530 | 23,084 | Valladas *et al*., 2017 |
| Cosquer | D | GifA14164/SacA39196 | Horse CHV057 | 19,890 | 130 | 24,275 | 23,590 | Valladas *et al*., 2017 |
| Cosquer | D | GifA98196 | HAF (Acid humic fraction) | 19,740 | 340 | 24,601 | 22,906 | Valladas *et al*., 2017 |
| Cosquer | D | GifA98186 | Horse CHV007 | 19,720 | 210 | 24,256 | 23,191 | Valladas *et al*., 2017 |
| Cosquer | D | GifA95135 | Megaceros MEG001 | 19,340 | 200 | 23,833 | 22,804 | Valladas *et al*., 2017 |
| Cosquer | D | GifA98188 | Stag CER001 | 19,290 | 340 | 24,025 | 22,489 | Valladas *et al*., 2017 |
| Cosquer | D | GifA92418 | Feline FEL001 | 19,200 | 240 | 23,710 | 22,539 | Valladas *et al*., 2017 |
| Cosquer | D | GifA14002/SacA37406 | Jellyfish shape nº 1 | 18,910 | 630 | 24,447 | 21,397 | Valladas *et al*., 2017 |
| Cosquer | D | GifA92416 | Horse CHV001 | 18,849 | 250 | 23,416 | 22,247 | Valladas *et al*., 2017 |
| Cosquer | D | GifA92417 | Horse CHV001 | 18,820 | 310 | 23,492 | 22,022 | Valladas *et al*., 2017 |
| Cosquer | D | *GifA92422* | *HAF (Acid humic fraction)* | *18,760* | *220* | *23,230* | *22,165* | Valladas *et al*., 2017 |
| Cosquer | D | GifA14003/SacA37407 | Horse CHV017 | 18,610 | 100 | 22,746 | 22,277 | Valladas *et al*., 2017 |
| Cosquer | D | GifA14168/SacA39200 | Penguin PIN003 | 18,590 | 110 | 22,757 | 22,216 | Valladas *et al*., 2017 |
| Cosquer | D | GifA92492 | Bison BIS001 | 18,530 | 190 | 22,840 | 21,921 | Valladas *et al*., 2017 |
| Cosquer | D | GifA14159/SacA39222 | Bison BIS004 | 18,200 | 110 | 22,365 | 21,795 | Valladas *et al*., 2017 |
| Cosquer | D | GifA92419 | Bison BIS001 | 18,010 | 200 | 22,355 | 21,303 | Valladas *et al*., 2017 |
| Cosquer | D | GifA96075 | Star shape SIG121 | 17,800 | 160 | 21,957 | 21,051 | Valladas *et al*., 2017 |
| Cosquer | D | GifA14001/SacA37405 | Jellyfish shape nº 2 | 17,120 | 80 | 20,909 | 20,426 | Valladas *et al*., 2017 |
| Cosquer | D | GifA14155/SacA39218 | Bison BIS001 | 16,590 | 90 | 20,287 | 19,723 | Valladas *et al*., 2017 |
| Cosquer | D | *GifA92423* | *HAF (Acid humic fraction)* | *16,390* | *260* | *20,451* | *19,148* | Valladas *et al*., 2017 |
| Cosquer | D | GifA14171/SacA39202 | Frandole SIG133 | 16,310 | 320 | 20,466 | 18,939 | Valladas *et al*., 2017 |
| Cosquer | D | GifA96101 | Median Jellyfish shape | 14,050 | 180 | 17,579 | 16,501 | Valladas *et al*., 2017 |
| Cosquer | C | *GifA14225/SacA39207* | *HAF (Acid humic fraction)* | *27,990* | *510* | *33,266* | *31,085* | Valladas *et al*., 2017 |
| Cosquer | C | GifA92350 | Ground (Scattered charcoal below FEL001) | 27,870 | 470 | 33,048 | 21,033 | Valladas *et al*., 2017 |
| Cosquer | C | GifA14227/SacA39209 | Ancient patina fireplace (soot under concrection) | 27,560 | 310 | 32,240 | 30,961 | Valladas *et al*., 2017 |
| Cosquer | C | GifA14166/SacA39198 | Ancient patina fireplace (soot under concrection) | 27,290 | 300 | 31,694 | 30,820 | Valladas *et al*., 2017 |
| Cosquer | C | *GifA14226/SacA39208* | *HAF (Acid humic fraction)* | *27,090* | *300* | *31,492* | *30,731* | Valladas *et al*., 2017 |
| Cosquer | C | GifA14165/SacA39197 | Fixed lamp (coal on a suspended floor) | 27,020 | 290 | 31,420 | 30,706 | Valladas *et al*., 2017 |
| Cosquer | C | GifA92349 | Ground charcoal bellow penguins | 26,360 | 440 | 31,195 | 29,586 | Valladas *et al*., 2017 |
| Cosquer | C | GifA92348 | Ground (Charcoal scattered below BIS001) | 20,370 | 260 | 25,255 | 23,916 | Valladas *et al*., 2017 |
| Cosquer | C | Ly 5528 | Fireplace | 18,400 | 440 | 23,369 | 21,186 | Valladas *et al*., 2017 |
| Cosquer | C | *GifA14224/SacA39206* | *HAF (acid humic fraction)* | *17,950* | *380* | *22,568* | *20,479* | Valladas *et al*., 2017 |
| Cosquer | C | GifA14170/SacA39201 | Power hammer in the concavity under penguins | 16,200 | 90 | 19,842 | 19,266 | Valladas *et al*., 2017 |
| Cosquer | C | GifA14161/SacA39193 | Recent patina combustion | 15,730 | 80 | 19,193 | 18,796 | Valladas *et al*., 2017 |
| Cosquer | C | Gif14153/SacA39216 | Ground (probably introduced by the sea) | 3990 | 90 | 4815 | 4160 | Valladas *et al*., 2017 |
| Pech-Merle | D | GifA95357 | Right Spotted Horse | 24,640 | 390 | 29,551 | 27,873 | Lorblanchet *et al*., 1995 |
| Pech-Merle | C | - | Reindeer metacarpian (sondage under Spotted horses) | 18,400 | 350 | 23,086 | 21,396 | Lorblanchet, 2010 |
| Pech-Merle | C | Ly-1200 | Charcoal (Sondage under Spotted Horses) | 11,380 | 390 | 14,285 | 12,431 | Lorblanchet, 2010 |
| Pech-Merle | C | Ly-1861 | Charcoal (sondage under *Frise Noir*) | 11,200 | 800 | 15,739 | 11,134 | Lorblanchet, 2010 |
| Pergouset | C | GifA96675 | Charcoal (in a ledge inside the decorated gallery) | 32,850 | 520 | 38,439 | 35,842 | Lorblanchet, 2010 |
| Gargas | C | GifA92369 | Fixed bone in the wall of the first hall | 26,860 | 460 | 31,713 | 29,950 | Clottes *et al*., 1992 |
| Gargas | C | GifA12190 | Fixed bone in the wall of the first hall | 26,680 | 490 | 31,531 | 29,707 | Le Guillou et al., 2018 |
| Cussac | C | GifA13150 | Charcoal (descent of the clay bridge, under a torch smear) | 25,150 | 210 | 29,704 | 28,714 | Jaubert *et al*., 2017 |
| Cussac | C | Beta156643 | Human bone (Locus 1) | 25,120 | 120 | 29,500 | 28,835 | Jaubert *et al*., 2017 |
| Cussac | C | Beta156644 | Human bone (Locus 2) - insufficient collagen | 15,750 | 50 | - | - | Jaubert *et al*., 2017 |
| Pileta | D | GifA98161 | Aurochs in the Breuil´s Sanctuary | 20,103 | 350 | 25,204 | 23,410 | Sanchidrian *et al*., 2001 |

**S3a Table. Chronological order information on radiocarbon dating of parietal art samples and their contexts (Direct Dating -D-; Contextual Dating -C-; Cal BP confidence level 95,4%. *Calibrated with OxCal*).**

| **Site** | **Lab code** | **Sample description** | **Age (yr BP)** | **Error** | **Age (Cal BP)** | | **Reference** |
| --- | --- | --- | --- | --- | --- | --- | --- |
|  |  |  |  |  | Max | Min |  |
| Isturitz | OxA-31096 | Bone in stratigraphical section from Salle des Phosphates | 27,010 | 250 | 31,364 | 30,750 | Unpublished |
| Gargas | *Unknown* | Units 6 and 7, located between 3 and 5 cm below a decorated stone (terminus *ante quem*) | Between 28,710 ± 200 and 30,738 ± 210 cal BP (intCal 13) | | | | San Juan-Foucher and Foucher, 2016 |
| Gargas | Ly1625GrA | Reinder antler (Cartaillac-Breuil locus, level 6) | 24,040 | 170 | 28,494 | 27,750 | Foucher, 2004 |
| Abri Laraux | Ly2101 | Bones in layer 3 (same level with portable art) | 21,950 | 350 | 27,104 | 25,634 | Pradel, 1979 |
| Abri Laraux | Ly1739 | Bone in layer 3 (same level with portable art) | 21,530 | 910 | 27,722 | 23,941 | Pradel, 1979 |
| Abri Pataud | OxA-686 | Bone in layer 3 (same level with portable art) | 24,500 | 600 | 30,075 | 27,573 | Bouchud, 1975 |
| Abri Pataud | OxA-165 | Bone in layer 3 (same level with portable art) | 24,440 | 740 | 30,392 | 27,408 | Bouchud, 1975 |
| Abri Pataud | OxA-164 | Bone in layer 3 (same level with portable art) | 24,250 | 750 | 30,292 | 27,218 | Bouchud, 1975 |
| Abri Pataud | OxA-685 | Bone in layer 3 (same level with portable art) | 23,200 | 500 | 28,423 | 26,410 | Bouchud, 1975 |
| Abri Pataud | OxA-163 | Bone in layer 3 (same level with portable art) | 23,180 | 670 | 28,706 | 26,096 | Bouchud, 1975 |
| Abri Pataud | GrN-4721 | Bone in layer 3 (same level with portable art) | 23,010 | 170 | 27,644 | 27,005 | Bouchud, 1975 |
| Abri Pataud | GrN-4506 | Bone in layer 3 (same level with portable art) | 22,780 | 140 | 27,439 | 26,689 | Bouchud, 1975 |
| Abri Pataud | OxA-599 | Bone in layer 3 (same level with portable art) | 21,740 | 450 | 27,142 | 25,191 | Bouchud, 1975 |
| Abri Pataud | GrN-1892 | Burnt bone in layer 3 (same level with portable art) | 21,540 | 160 | 26,102 | 25,543 | Bouchud, 1975 |
| Abri Pataud | GrN-1864 | Burnt bone in layer 3 (same level with portable art) | 18,470 | 280 | 22,991 | 21,684 | Bouchud, 1975 |
| Parpalló | Birm-520 | Bone 7.25-7.75m pre-Gravettian | 20,170 | 380 | 25,324 | 23,425 | Aura & Villaverde, 2014 |
| Parpalló | BM-859 | Bone 6.5-7m Solutrean | 20,490 | 900 | 26,818 | 22,735 | Aura & Villaverde, 2014 |
| Parpalló | BM-861 | Bone 5-4,75m Upper Solutrean | 18,080 | 770 | 23,840 | 20,096 | Aura & Villaverde, 2014 |
| Parpalló | Birm-521 | Bone 4,25-4m Upper Solutrean | 17,896 | 340 | 22,448 | 20,815 | Aura & Villaverde, 2014 |

**S3b Table. Chronological order information in radiocarbon dating of portable art contexts (Cal BP confidence level 95,4%. *Calibrated with OxCal*).**
