# Supplementary material for: Redefining shared symbolic networks during the Gravettian in Western Europe: New data from the rock art findings in Aitzbitarte caves (Northern Spain)
Source: PLoS One. 2020 Oct 28;15(10):e0240481. doi: 10.1371/journal.pone.0240481 (PMC7592797; doi:10.1371/journal.pone.0240481)
Supplement: S1 File — (DOCX) [file pone.0240481.s004.docx]

**U/Th speleothem dating in Aitzbitarte caves**

Seven speleothem samples were taken for U/Th dating, in order to reconstruct the engravings passage morphology for the time where it was decorated. Two samples belong to a narrow meandering passage that give access to the decorate area of Aitzbitarte III cave, and they are coating a decametric boulders that make the passage smaller and relatively difficult to access. The aim of these speleothem samples was to reveal if the passage was as wide as nowadays, or on the contrary, it was wider when the Gravettian artist visited the cave. However, after extracting from the field the best possible samples, the quality of the crystal was not good enough (porous, presence of clastic sediment, and diagenetic crystal fabric), showing an open system, and the CENIEH Uranium Series Laboratory was not able to obtain a reliable age from them (S1 Table).

| **Sample** | **AIT_III-1ext** | **AIT_III-1int** |
| --- | --- | --- |
| **Crystal fabric** | Microsparite | Dendritic porous |
| **U (µg/g)** | 0.039 ± 0.002 | 0.056 ± 0.003 |
| **Th (µg/g)** | 0.350 ± 0.002 | 0.462 ± 0.002 |
| **^230^Th/^232^Th x10^-6^** | 7.3 ± 0.004 | 4.5 ± 0.01 |
| **δ^234^U** | 516 ± 3 | 444 ± 3 |
| **^230^Th/^238^U** | 3.988 ± 0.004 | 2.240 ± 0.006 |
| **Age ^230^Th (BP)** | - | - |

**S4 Table. Sample crystal fabric (according to Frisia (2015) summary) and result of the U/Th dating of speleothems in Aitzbitarte Cave III. The analyses were performed in the CENIEH Uranium Series Laboratory (Spain).**

The other five samples were taken from the collapsed blocks deposit that fills the passage along 20 m between the entrance of Aitzbitarte IX and the engraved area (Figure 4). Three samples (AIT_IX-1, AIT_IX-2 and AIT_IX-3) belong to the outer part of the collapse, which is accessed from the current Aitzbitarte IX entrance. The other two samples (AIT_IX-4 and AIT_IX-5) belong to the inner part of the collapse, which is currently accessed from the decorated passage (Aitzbitarte III cave entrance, Figure 4). Regarding AIT_IX-4 speleothem sample, being an *in situ* column, two internal samplings where performed, one from the centre (AIT_IX-4int) and the other from the outer part (AIT_IX-4ext), to know its initial and final ages. In the end, the aim of these samples was to clarify if the collapse was there when the passage was decorated, and therefore, if Palaeolithic humans entered this area from Aitzbitarte III cave entrance, or on the contrary, the collapse did not still exist and they entered the place from Aitzbitarte IX cave entrance.

In this case, the crystal quality of AIT_IX-2, AIT_IX-4ext, AIT_IX-4int and AIT_IX-5 samples show a primary crystal fabric and a close system (Table 1), giving reliable results. For sample AIT_IX-1, whether it shows a primary crystal fabric, it contain dissolution features that could imply an open system (Muñoz-García et al., 2012), therefore the real age could be somewhat different from the obtained. And finally, sample AIT_IX-3 display an open system features and poor crystal quality, so the CENIEH Uranium Series Laboratory was not able to obtain a reliable age (Table 1).

To conclude, samples AIT_IX-1 and AIT_IX-2, which were taken from the upper part of the collapsed blocks, reveal the oldest ages of all samples, being AIT_IX-2 the oldest with 125.712 ± 1.333 ka (Table 1). Therefore, the blocks under these speleothems should be there previously, and so we interpret that Aitzbitarte IX entrance was already blocked by the collapse when the engraving hall was decorated. However, the speleothem dated from the inner part of the collapse reveals Holocene ages, meaning that in the decorated hall, the last fallen blocks could occur after the Palaeolithic human occupation, and so the hall morphology could be slightly different.

**References**

Frisia, S., 2015. Microstratigraphic logging of calcite fabrics in speleothems as tool for palaeoclimate studies. International Journal of Speleology 44, 1-16.

Muñoz-García, M.B., López-Arce, P., Fernández-Valle, M.E., Martín-Chivelet, J., Fort, R., 2012. Porosity and hydric behavior of typical calcite microfabrics in stalagmites. Sedimentary Geology 265-266, 72-86.
