# Supplementary material for: Redefining shared symbolic networks during the Gravettian in Western Europe: New data from the rock art findings in Aitzbitarte caves (Northern Spain)
Source: PLoS One. 2020 Oct 28;15(10):e0240481. doi: 10.1371/journal.pone.0240481 (PMC7592797; doi:10.1371/journal.pone.0240481)
Supplement: S2 File — (DOCX) [file pone.0240481.s005.docx]

Virtual visit to the caves in:

<https://kuula.co/post/7T61g/collection/7PYHb>
